# A Comprehensive Review of the Biological Activities of Medicinal Metal Complexes Synthesized From Quinoline Scaffolds

**DOI:** 10.1155/bca/3133615

**Published:** 2025-02-11

**Authors:** Sabikeh G. Azimi, Neda Shakour, Ghodsieh Bagherzade, Mohammad Reza Saberi, Hosseinali Azimi, Mehdi Moosavi F.

**Affiliations:** ^1^Department of Chemistry, Faculty of Sciences, University of Birjand, Birjand 97175-615, Iran; ^2^Department of Medicinal Chemistry, School of Pharmacy, Mashhad University of Medical Sciences, Mashhad, Iran; ^3^Department of Research and Development, Parthkimia Pharmaceutical Co., Gorgan, Iran; ^4^Department of Research and Development, Golestan Science and Technology Park, Gorgan, Iran; ^5^Department of Organic Chemistry, Faculty of Chemistry, University of Mazandaran, Babolsar, Iran

**Keywords:** biological activity, DNA binding, drug performance, *in silico* studies, medicinal metal complexes, quinoline scaffold

## Abstract

The compelling attributes of quinoline scaffolds in medicinal compounds have garnered considerable attention from researchers, due to their notable biological efficacy, biocompatibility, and distinctive photophysical properties. Quinoline complexes, in particular, have emerged as significant entities, demonstrating a wide array of medicinal properties, including antibacterial, antifungal, antiviral, anticancer, anthelmintic, anti-HIV, antioxidant, antituberculosis, and antimalarial activities. In addition, they showed promise in photodynamic and neurological studies, along with strong DNA-binding capabilities. In recent years (2010–2023), substantial progress has been made in understanding quinoline complexes. Key aspects such as the lipophilicity, of metal complexes, enzymatic drug degradation factors influencing inhibition, drug performance, disruption of target cell growth, and their impact on DNA have been thoroughly investigated. Researchers have employed advanced methodologies including fluorescent imaging, determination of MIC and IC_50_ values, hydrodynamic and spectrophotometric techniques, in silico and in vitro studies, and cytotoxicity assessments using the MTT method, to significantly enhance our understanding of these complexes. Recent findings indicated that the interaction of quinoline complexes with viral proteins and their ability to disrupt enzyme-viral DNA relationships have made them powerful therapeutic agents for severe diseases including cancer, AIDS, and coronaviruses, as well as various neurological and microbial infections. It is anticipated that these explorations will lead to effective advancements in therapeutic strategies within modern medicine.

## 1. Introduction

**Figure 1:**
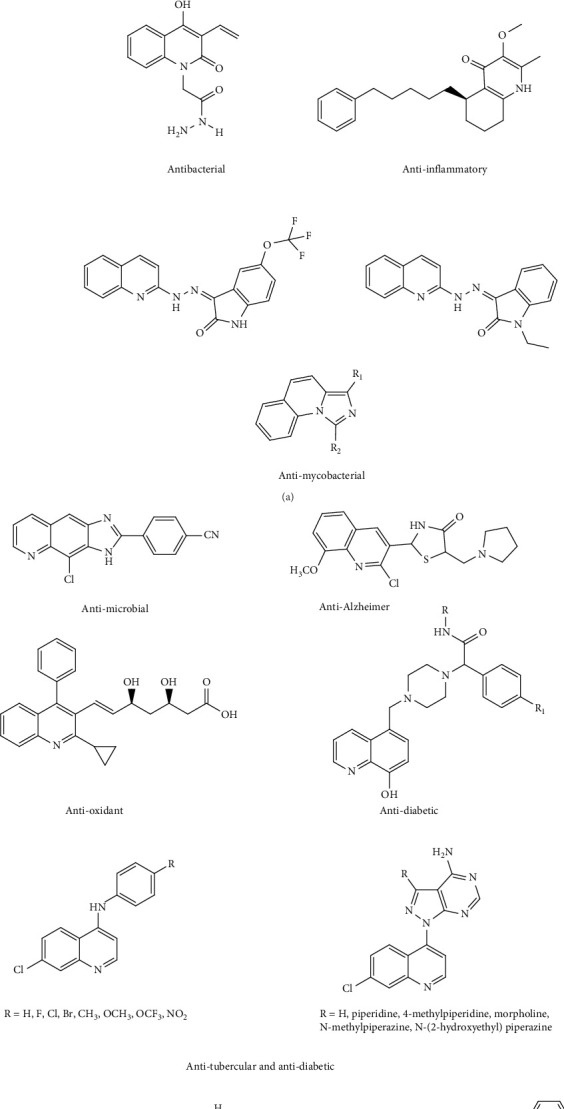
Structures of drug molecules containing quinoline scaffold.

One of the most attractive and important chemical motifs with high medicinal potential is the quinoline molecule. This molecule is found in medicinal and natural alkaloids such as Cinchona alkaloids, Echinopsine, Vasicine, Fabrifugine, Cosparin, and Galipin. Quinoline plays a crucial role in drug development by participating in electrophilic and nucleophilic substitution reactions, forming quinoline derivatives with cytotoxic activity. Due to its diverse chemical and medicinal properties, quinoline is utilized in the production of various drugs, including: anticancer [1–7], antioxidant [8, 9], antitubercular [10, 11], antidiabetic [12–16], anti-Alzheimer [17], anti-COVID-19 [18–23], anti-plasmodial [24–26], anti-inflammatory [27–30], antimycobacterial [31–33], antimicrobial [34, 35], anticonvulsant [36–38], antibacterial [39–41], and anti-HIV-1 [42–45]. These drugs have been used as potent inhibitors of protein tyrosine and kinase [46, 47] and in the production of chemical sensors [48–50]. See the structure of compounds in Figure 1. The importance of quinoline-containing metal complexes as biologically active pharmaceuticals has increased significantly. Platinum complexes [51], one of the oldest metal-based pharmaceuticals, exhibit proteasome inhibitor properties [52–54], direct apoptosis induction [55], topoisomerase inhibition [56], or redox imbalance [57]. Today, metal complexes are used in the pharmaceutical industry, such as structural and therapeutic probes, tracking agents, and sequence-specific binding agents [58–62]. In the formation of these metal complexes, transition metals have been favored over other main group elements due to their ability to undergo oxidation and reduction. These compounds can disrupt cellular homeostasis by oxidation–reduction sensitive processes [63]. In addition, they can inhibit the cell wall formation by disrupting cellular respiration, leading to cell death. Alternatively, they can prevent cell proliferation by inhibiting the DNA gyrase enzyme [64]. The formation of metal complexes enhances drug efficacy through the transfer of electrons from the ligand to the metal. This enhances lipophilicity, allowing the complexes to more easily traverse the lipid cell membrane of bacteria. Furthermore, complexes of vital metal ions can target DNA by interacting with and forming covalent bonds with the DNA phosphate backbone. Noncovalent interactions, including electrostatic and groove binding, can also occur with the central metal ion, ultimately inhibiting the activity of cancer cells [65]. Several factors significantly influence the biological activity of quinoline metal complexes. These include coordination geometry [66], polarizability, and hardness/softness of the coordinated species and their effects on coordination [67], standard reduction potential of the central atom in the formed complex [68], hydrogen bonding ability of the complexes, high crystal field stabilization energy (CFSE), increased stability constant [69], electric charge, and size of the central atom [70]. These factors play a crucial role in the mechanism of drug action by influencing the stability of the complex and its binding affinity to DNA. This article aims to review the biological studies of quinoline metal complexes conducted from 2010 to 2023, focusing on their antibacterial, antifungal, antiviral, anticancer, antiworm, anti-HIV, antioxidant, antituberculosis, and antimalarial activities, as well as their photodynamic activity and neurological impacts. This review comprehensively investigates and discusses the factors affecting these activities, highlighting the vital importance of these complexes. We hope this review will inspire new ideas and innovations in this scientific field.

## 2. Materials and Methods

This academic investigation compiled information from reputable scholarly databases, including PubMed, Science Direct, Google Scholar, and Scopus. Our data collection covered the period from 2010 to 2023. The specified search terms used were “biological activity” or “biological products,” “metal complexes,” and “quinoline.”

## 3. Biological Activity

### 3.1. Anticancer Activity

**Figure 2:**
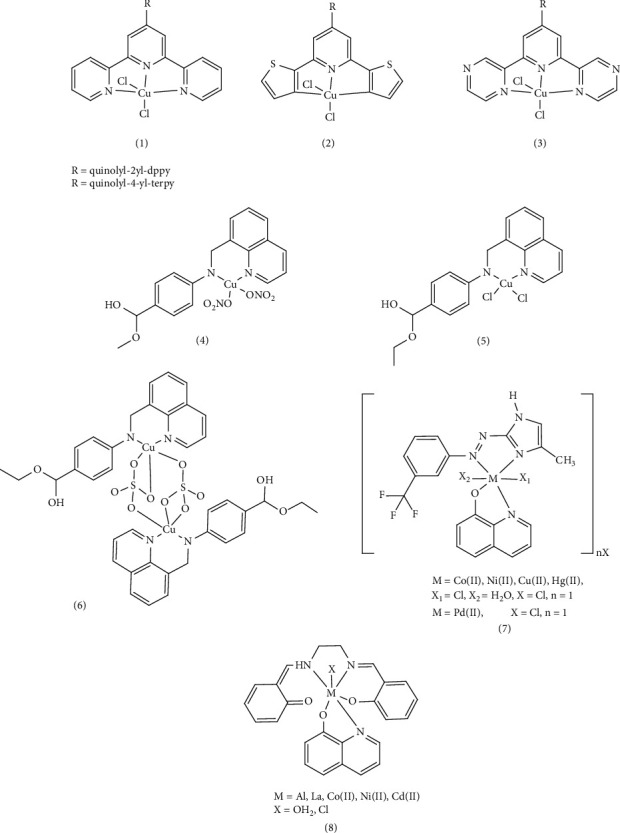
The chemical structure of quinoline metal complexes (1–8) with anticancer activity.

Katarzyna et al. synthesized 2,2′:6′,2″- terpyridine (terpy), 2,6-di(thiazol-2-yl) pyridine (dtpy), and 2,6-di(pyrazin-2-yl) pyridine (dppy) ligands and their copper (II) complexes (1–3). The antiproliferative and catalytic properties of five coordinate complexes (CuCl_2_ (κ^3^-L)) were studied, along with the effects of several triimine skeletons, such as terpy, dtpy, and dppy, and n-quinolyl substitution. Studies on colon (HCT116) and ovarian (A2780) cancer cells found that Cu (II) terpy and Cu (II) dppy complexes significantly suppressed the antiproliferative impact in normal fibroblasts. One of the primary mechanisms potentially causing apoptosis in cancer cells is the increased production of reactive oxygen species (ROS). For this reason, ROS production in HCT116 cells exposed to Cu (II) quinolyl-2yl-dppy (complex, 1,) and Cu (II) quinolyl-4-yl-terpy (complex, 1,) for 48 h was investigated. By chelating intracellular copper and participating in the redox cycle, these complexes led to the production of ROS and hydroxyl radicals. A significant increase of 97% in cellular ROS was observed for Cu(II)quinolyl-2yl-dppy (Complex 1) and a 31% increase for Cu(II)quinolyl-4-yl-terpy (Complex 1) [71]. These complexes, such as anticancer drugs such as cisplatin, may prevent DNA replication and cause cell death by covalently binding the central metal with DNA, replacing some ligands attached to the central atom with a part of DNA, or through the noncovalent interaction groove to bond with DNA [72]. In another study, three copper complexes ([Cu(L_1_) (NO_3_)_2_] (complex, 4,), [Cu(L_2_) Cl_2_] (complex, 5,), and [Cu(L_2_) SO_4_]_2_H_2_O ((complex, 6,) derived from quinoline Schiff base ligands (a mixture of quinoline-8-carbaldehyde with 4-amino-benzoic acid methyl ester (L_1_) or 4-amino-benzoic acid ethyl ester (benzocaine) (L_2_)) were designed and prepared. Cytotoxicity assays indicated that these complexes exhibited substantial cytotoxicity, possibly due to multiple mechanisms of oxidative damage, particularly for Complex 6. Compared to normal HL-7702 cells and three other tumor cell lines (Hep-G2, NCI-H460, and MGC80-3), Complex 6 demonstrated the highest toxicity with IC_50_ values significantly lower than cisplatin and ligands. Considering that the sulfate group is a better leaver than nitrate and chloride groups, and Complex 6 likely prevents DNA replication by replacing the sulfate group with a fragment of DNA with covalent binding, contributing to its higher anticancer properties compared to other complexes. These copper complexes exhibited low cytotoxicity on normal HL-7702 cell lines, suggesting the natural selectivity for HeLa cells compared to normal cells [73]. Widad et al. successfully synthesized new azo ligands (8-hydroxyquinoline (8-HQ)) and Complex 7 ([ML (8-HQ) Cl_2_] H_2_O, [ML (8-HQ)] Cl; M= Co (II), Zn (II), Cu (II), Ni (II), and Pd (II)). They then investigated the biological effects and cytotoxicity inhibition results of the Pd (II) complex on human breast cancer cell lines (MCF-7 and AMJ-13) and normal cells (HBL). The toxicity effect of Pd L_1_ (8-HQ)] Cl_2_ on prostate cancer cells was also investigated. Based on the experimental results, an IC_50_ = 151.5 was observed for prostate cancer cells (CP_3_) and an IC_50_ = 243.7 for healthy liver cells (WRL-68). These results suggest that at a concentration of 100 μg/mL, the complex destroys half of prostate cancer cells, indicating a strong potential for inhibiting prostate cancer cell growth. In conjunction with Pd (II) complexes, this efficacy is likely due to the square planar geometry of the palladium complex, which is proven based on electron transfers and its electrolytic nature. The square planar geometry allows for better binding to DNA compared to other complexes with octahedral geometry resulting in enhanced anticancer properties. These results also demonstrated that complexes have a greater effect on action than ligands alone [74]. In another study, Badriah et al. investigated and synthesized mixed ligand metal complexes (MSQ) (Complex 8), containing salin (S) and 8-hydroxyquinoline (Q) with (AlCl_3_.6H_2_O), (CdCl_2_.H_2_O), (LaCl_3_.7H_2_O), (NiCl_2_.6H_2_O), and (CoCl_2_.6H_2_O) salts. The anticancer activity of these synthetic compounds was assessed, based on cytotoxicity studies against aggressive human breast cancer cell lines (MDA-MB231) and liver cancer cell line (Hep-G2). The salen ligand and complexes, particularly CdSQ, exhibited high cytotoxicity with low IC_50_ values compared to the reference drug cisplatin. In addition, molecular docking studies were conducted using MOE 2014 and Gaussian 09 software in active site of breast cancer oxide reductase (PDB ID: 3HB5), the Cd (II) metal complex had the lowest binding energy compared to the ligand [75] (Figure 2).

In another study, Hessah et al. presented a straightforward method for producing a new series of Zn (II), Pd (II), VO(II), and Cr (III) and Complex 9 derived from BSQ = 4-Bromo-2-(quinolin-8-yliminomethyl). The antiproliferative activities of these compounds were evaluated against breast cancer cells (MCF- 7) and cell lines HCT-116 and HepG-2. Results showed that these complexes exhibited greater efficiency than the free ligand. Furthermore, their cytotoxicity was lower than that of the standard drug (vinblastine). Based on the observed changes in the characteristic band of the complexes, the researchers demonstrated an interaction with DNA and observed an intercalative contact. Investigation of the binding potential of the complexes, including studies of changes in the electronic spectra and observation of hyperchromic shifts, indicated both binding and electrostatic interactions between the complexes and DNA. The higher binding constant values obtained for the zinc complex suggested a stronger binding affinity, potentially explaining its enhanced cytotoxicity in destroying cancer cells and inhibiting DNA transcription. This strong binding may be attributed to the desirable square pyramidal geometry of this complex and its noncovalent interaction with DNA. Furthermore, computer simulations were conducted using Gaussian 09, Pharmit link, and MOE-docking with proteins (PDB ID: 4ewp, 6isv, 7kcd). These simulations demonstrated that the BSQPd and BSQZn complexes exhibited the most favorable interactions with the lowest energy values, supporting their potential therapeutic applications [76]. Bastien et al. investigated the anticancer activities of the 8-hydroxyquinoline-copper complex on the leukemic cell line RAW264.7 using two-dimensional electrophoresis techniques. Their study focused on the ubiquitin/proteasome pathway, the mitochondrion and cell adhesion-cytoskeleton pathway, and carbon metabolism. The results revealed that the hydroxyl quinoline–copper complex can reduce free glutathione levels alter the actin cell structure and target cancer stem cells. Further proteomic studies on the effects of the copper 8-hydroxyquinoline complex, oxidative stress response and the cytoskeleton of leukemia cells revealed a pleiotropic effect. This suggests that the complex not only reduces cancer cells' resistance but also enhances drug toxicity, potentially offering a promising therapeutic strategy for treating leukemia. Therefore, copper complexes demonstrated selective cytotoxicity against cancer cells, leading to their reduction. This indicates a significant impact of copper complexes on the proteome compared to hydroxyquinol [77]. In a separate study, Zn (II), Al (III), Cu (II), and Ru (II) quinoline complexes with fluorescent properties and biological activities were synthesized. Complex 10b exhibited superior anticancer properties against the breast cancer cell line (MCF-7) compared to cisplatin and cyclophosphamide. Complex 10a, possessing the highest fluorescence quantum efficiency, holds potential as a biological marker. Evaluations of myelotoxicity, hemostasis, and hepatotoxicity of these synthetic substances revealed no significant differences in red blood cells and platelets, suggesting no myelotoxic effect [78].

The effects of synthesized Complex 11, including [Zn_2_ (^MeOOC^L^COO^) (CH_3_COO)_2_] and [Cu_2_(^MeOOC^L^COO^)(CH_3_COO)_2_], on the cytotoxicity of ^EtOOC^HL^COOEt^ were investigated in three human cancer cell line: A549 (non–small-cell lung carcinoma), CH1 (ovarian carcinoma), and SW480 (colon adenocarcinoma), using fluorescence microscopy. These compounds demonstrated effects on both the endoplasmic reticulum and lysosomes, contributing to their cytotoxicity activity. All complexes displayed low IC_50_ values and significant antiproliferative activity against the three human cancer cell lines (A549, CH1, and SW480). Interestingly, A549 exhibited greater resistance to chemotherapy drugs, with IC_50_ values 13 times higher than those of CH1 cells. The zinc (II) complex showed superior cytotoxicity, better solubility, and lower IC_50_ values in a compatible environment compared to metal-free ligand and copper (II) complex [79]. This difference may be attributed to the Jahn–Teller effect. The copper (II) ion in the octahedral geometry is susceptible to Jahn–Teller distortion. Due to the asymmetrical electron arrangement, the regular octahedral structure with Oh symmetry lacks stability. Consequently, these complexes deviated from the regular octahedral state, resulting in a reduction of symmetry and adoption of a tetragonal arrangement (tetrahedron or square pyramids) for increased stability. This Jahn–Teller effect may enhance the availability of copper metal for cells, potentially reducing its toxicity. Ammar et al. successfully synthesized new Schiff base ligands [1, 3‐diphenyl‐1H‐pyrazole‐5‐yl) methylenedehydrazineylquinoline (H‐DPPMHQ)] and Complex 12 of Co (II), Ni (II), Cu (II), and Zn (II) and investigated their anticancer properties. Anticancer activities were evaluated against human breast cancer (MCF-7) and lung cancer (A549) cell lines using the MTT method, with results expressed based on IC_50_ (maximum inhibitory concentration) values. The order of cytotoxicity of the synthetic complexes was reported as Cr (III) > (H-DPPMHQ) > Co (II) > Ni (II) > Cu (II) > Zn (II). The dimeric chromium (III) complex displayed higher cytotoxicity than other complexes for both cancer cell lines, potentially due to its strong binding affinity to the DNA of cancer cells, leading to dysfunction. Cytotoxicity may be linked to the stability of synthetic complexes. The larger central atom size, generally results in less stable complexes, increasing the likelihood of DNA binding. This, in turn, can inhibit DNA replication and lead to cell death [40]. Shakir et al. investigated the antiproliferative activity of Compound 13, including Mn (II), Co (II), Ni (II), Cu (II), and Zn (II), with a quinoline Schiff base ligand ((E)-N-(furan-2-yl methylene) quinolin-8-amine, derived (L)) on three cell lines: MDA-MB-231 (breast carcinoma), KCL22 (blood lymphoid carcinoma), HeLa (cervical carcinoma), and normal cells (PBMC). Metal chelates exhibited greater activity compared to their corresponding ligands. Notably, the Mn (II) complex demonstrated particularly promising activity against all cancer cell lines tested, with IC_50_ < 2.10 μM. This enhanced efficacy is attributed to several potential cytotoxic factors, including kinetic and thermodynamic stability: The Mn (II) complex possesses a small size, high charge density, and favorable coordinated geometry, contributing to its stability.

**Figure 3:**
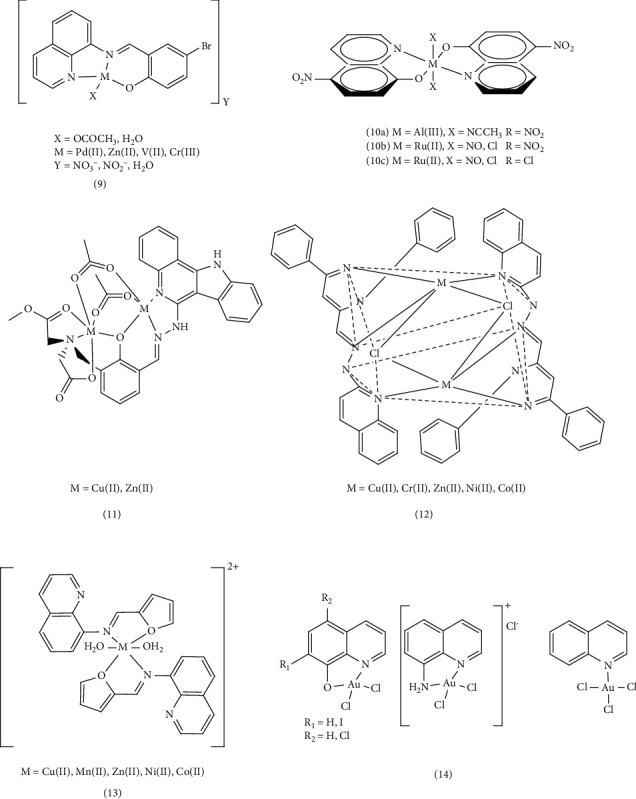
The chemical structure of quinoline metal complexes (9–14) with anticancer activity.

• Redox potential: The complex's redox potential plays a crucial role in its cytotoxic activity.
• Hydrogen bonding ability: The Mn (II) complex's ability to form hydrogen bonds further enhances its interaction with cellular targets. Furthermore, the stronger binding affinity of the ligand to DNA, compared to other complexes, led to a reduction in DNA replication and ultimately, cancer cell death. Mn (II) forms a more unstable complex due to having the lowest electrode potential of the standard reduction of the central atom and the size of the central atom compared to other central atoms. The more unstable the complex, the stronger the binding to DNA and the greater the cytotoxicity is [80]. The synthesis and antiproliferative properties of Au (III) Complex 14 with quinoline ligands were investigated against several human tumor cell lines. With the investigation of antiproliferative activities on human tumor cell lines such as A427 (lung cancer cell line), LCLC-103H (large cell lung cancer), SISO (uterine adenocarcinoma), and 5637 (human bladder), some of these complexes, such as [AuCl_2_(8) -O-quinoline] and [AuCl_2_(5-Cl-8-O-quinoline)] and [AuCl_2_(8-NH_2_-quinoline)] showed higher antiproliferative activity than cisplatin, particularly against lung cancer cell line (A427) [81]. This was attributed to the faster ligand exchange rate of Au (III) complexes compared to Pt (II) complexes. This allows them to bind to DNA more easily, preventing cell proliferation using covalently binding with DNA, such as replacing the unstable part of the complexes with a nitrogenous base of DNA (guanine N7) [69]. (Figure 3).

**Figure 4:**
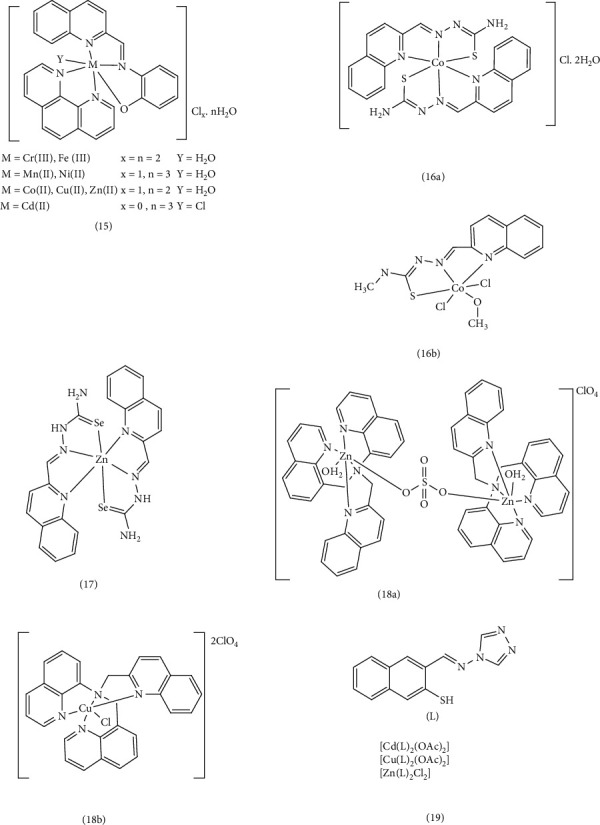
The chemical structure of quinoline metal complexes (15–19) with anticancer activity.

The researchers synthesized a quinoline ligand Schiff base which is a mixture of quinoline-2-carboxaldehyde with 2‐aminophenol (HL)) and its corresponding metal complexes of Mn (II), Fe (III), Ni (II), and Cu (II) and 1,10‐phenanthroline (Complex 15). They evaluated the anticancer activity of these metal complexes against breast cell lines (MCF-7). The IC_50_ values was as follows this order: Cu(II) > Cd(II)≈Ni(II) > Co(II) > Cr(III))≈Fe(III)≈Mn(II) > 1,10-phenanthroline > Zn(II). This indicates that the Cu (II) complex exhibited the highest anticancer activity, while the Zn (II) complex showed the lowest. The researchers also investigated the anticancer activity of these metal complexes against colon cancer cell lines (HCT-116). The IC_50_ values for HCT-116 cells followed this order: Zn(II) > Cr(III) > Cu(II) > Fe(III) > 1,10-phenanthroline > Co(II) > Ni(II) > Cd(II) > Mn(II). The chelation process led to increased activity compared to the Schiff base ligand alone. This enhancement is likely due to several factors, including the metal ion's nature and charge, the resulting complex's geometric structure, and its increased ability to permeate the cell membrane. While Mn (II) has a larger size than other central metals, it forms a less stable complex. This instability, however, allows for easier binding to DNA, resulting in higher cytoxicity, particularly against colon cell line (HCT-116) [46]. The researchers also synthesized two thiosemicarbazone complexes of cobalt and investigated their interactions with DNA and their cytotoxic properties. They examined the in vitro cytotoxicity of two Co (II) complexes against human lung adenocarcinoma (A-549/CDDP) and human breast adenocarcinoma (MCF-7) cell lines, comparing their activity to cisplatin. Both Co (II) complexes demonstrated significant activity against these cell lines, particularly Complex 16b. When the complexes were added to calf thymus DNA (CT-DNA), Complex 16b showed a greater increase in the hyperchromicity compared to Complex 16b at wavelengths of 298 and 400 nm. This suggests that Complex, 16b binds to DNA through a nonclassical internal binding mechanism, such as binding to the DNA groove. The stronger bond between the soft head (Co^2+^) and the soft head (S^2−^) in the Complex 16a makes it less easily separated. This suggests that Complex 16a can only engage in noncovalent interactions, including groove binding in the major or minor groove of DNA. In contrast, Complex 16b is more easily hydrolyzed, allowing chlorine to be replaced, which may contribute to its enhanced DNA binding. In Complex 16b, chlorine is replaced by water, leading to the formation of a covalent bond between the central metal and DNA. This is supported by the correlation between the binding constants (*K*_*b*_) and the degree of hyperchromicity, which indicates that Complex 16b exhibits stronger binding to DNA compared to Complex 16a. This stronger binding likely contributes to the significantly cytotoxicity observed for Complex 16b compared to Complex 16a the synthetic ligands and even cisplatin [82]. Filipović et al. synthesized and designed a series of 2-quinoline carboxaldehyde selenosemi carbazone ligands (Hqasesc) and their corresponding zinc (II) Complex 17. They investigated the interaction of these complexes with DNA/HSA using molecular docking, as well as absorption and emission spectral methods. Their studies revealed that the [Zn (Hqasesc)_2_] (ClO_4_)_2_ complex did not directly bind to DNA but exhibited a high tendency to be located in the minor groove of DNA. The strong connection in Complex 17 suggests that it cannot be easily separated and can only interact with DNA through noncovalent interactions, such as groove binding in the major or minor groove. The disappearance of the shoulder and peak bands at 296 nm, accompanied by the appearance of the hyperchromic absorption band at 343 nm and a blue shift of 10 nm at the maximum concentration, proves evidence for the interaction of the complex with CT-DNA. Furthermore, experimental results indicated that [Zn (Hqasesc)_2_] (ClO_4_)_2_ complex exhibits a high affinity toward human serum albumin (HSA) due to its pharmacokinetic properties. In evaluating the antitumor activity of the ligand and Zn (II) complex against pancreatic adenocarcinoma cells (AsPC-1) and acute monocytic leukemia cells (THP-1), both compounds demonstrated the ability to induce strong apoptosis in THP-1 cells. However, they exhibited less activity against the AsPC-1 cell line [83]. Jing Lu et. al synthesized two new quinoline complexes, 18a and b, and investigated their interaction with calf thymus deoxyribonucleic acid (CT-DNA) under physiological conditions using fluorescence spectroscopy, UV absorption, and gel electrophoresis. To examine DNA binding, the complexes were found to bind to CT-DNA via an intercalative mod, with partial insertion of aromatic heterocyclic rings between DNA base pairs, leading to p-p overlap interactions. This interaction resulted in a visible DNA cleavage under UV-A light at 365 nm. The increase in the CT-DNA concentration, hypochromism and red shift in the maximum peak indicating efficient binding of CT-DNA to the complex through an intercalative mode. In the in vitro cytotoxicity evaluations using the MTT method revealed that both complexes, 18a and 18b, exhibited a potential antiproliferative activity against four human tumor cell lines: 7404, HeLa, MCF-7, and HepG-2. Notably, the Cu (II) complex displayed particularly low IC_50_ values (6.867 and 5.957 μM) compared to cisplatin (IC_50_ value: 3.92 and 9.92 μM), suggesting its potential as an effective and potent anticancer drug [84]. A series of metal Complexes 19, (M = Cd (II), Cu (II), and Zn (II)), incorporating a quinoline Schiff base ligand (3-[(E)-(4H-1,2,4-triazol-4-yl imino) methyl] quinoline-2-thiol (L)), were synthesized. Subsequent investigations into the interaction of these complexes with CT-DNA revealed a decrease in the energy of the π ⟶ π^∗^ transition, along with notable hypochromism and a red shift. These findings, coupled with the intrinsic binding constant (*K*_*b*_) values, indicated an intercalative mode of interactions between the metal complexes and DNA. The strong interactions observed prompted further investigation of breast anticancer activity against estrogen receptor (PDB ID: 2IOK) and EGFR tyrosine kinase (PDB ID: 2a91), primary growth factors in breast cancer cells. This investigation utilized docking experiments with HEX 4.2 software. The metal complexes, particularly the copper complex, exhibited the lowest binding energy (E-total-30.84 kjmoL−1) compared to the standard drug Toremifene, suggesting a significant ability to inhibit breast cancer cells [52] (Figure 4).

Another study examined the anticancer properties, antifungal activities, and DNA binding of quinoline complexes in vitro. Based on the change in absorption values and the calculated intrinsic binding constants (*K*_*b*_) calculated for the complexes, the Co (II) complex exhibited higher *K*_*b*_ values than the Ni (II) complex. This observation suggests stronger binding of intercalators in the Co (II) complex, potentially due to the larger size of Co (II), the instability of this complex compared to Ni (II), and the stronger connection of this compound in DNA grooves. It is likely that the complexes interact with CT-DNA through groove binding with a lesser tendency to intercalation. MTT cytotoxicity studies conducted on human breast adenocarcinoma (MCF-7) cells revealed that the Ni (II) Complex 20 exhibited greater cytotoxicity than the Co (II) complex. The Ni (II) complex inhibited the proliferation of cancer cells by inducing apoptosis through the expression of Caspase-3, the production of ROS, and DNA damage [85]. Chen et al. successfully synthesized oxoglaucine (OG) and four Complex 21, M(OG)_2_(H_2_O)_2_] (ClO_4_)_2_ with M representing Mn(II), Co(II), Zn(II), and Au(III). They measured cytotoxicity on different tumor cell lines (BEL7404, A549, HeLa, and MCF-7) using the MTT method. The metal-OG complexes demonstrated higher cytotoxicity than OG, highlighting a positive synergistic effect [86]. The synthesis of Co (II), Cu (II), and Zn (II) complexes (Complexes 22) of three newly synthesized 6-methyl-2-oxo-quinoline-3-carbaldehyde thiosemicarbazone complexes was achieved. The cytotoxicity of these compounds was evaluated in vitro using the MTT method to determine IC_50_ values on various tumor cell lines, including SK-OV-3, BEL-7404, HeLa, Hep-G2, and MGC80-3, as well as normal liver cells (HL-7702). Cisplatin was served as a positive control in the study. It was observed that all three complexes exhibited lower inhibitory effects on normal human liver HL-7702 cells compared to cisplatin. However, these complexes displayed good cytotoxicity against other cancer cell lines, such as SK-OV-3 and BEL-7404. Notably, the complexes exhibited the highest inhibitory activity compared to the free ligand. Among the complexes, the copper complex demonstrated superior performance on MGC80-3 and SK-OV-3 cells compared to the other complexes and cisplatin, as evidenced by lower IC_50_ values. Subsequent investigations evaluated the binding mode between the complexes and DNA by examining UV-Vis, fluorescence, and CD spectra, as well as gel mobility shift assays. This study revealed that the copper complex, through mitochondrial dysfunction, induces apoptosis in MGC80-3 cells, leading to cell cycle arrest in the S phase, the production of high levels of ROS compared to the control cells in MGC80-3, and ultimately to apoptosis or cell death [55].

### 3.2. Antimicrobial Activity

This section highlights the research on quinoline metal complexes, emphasizing their potential for antimicrobial activity. The research groups discussed synthesized various quinoline-based compounds and their metal complexes, investigating their biological activities and underlying mechanisms. In other in vitro studies, the antimicrobial activity of quinoline metal complexes against Gram-positive bacteria *Bacillus subtilis (B. subtilis)* and *Staphylococcus aureus (S. aureus)*, Gram-negative *Pseudomonas aeruginosa (P. aeruginosa)*, and *Escherichia coli (E. coli)*, and two fungi (*C. albicans* and *A. flavus*) were investigated. The results of these tests are summarized in this section.

**Figure 5:**
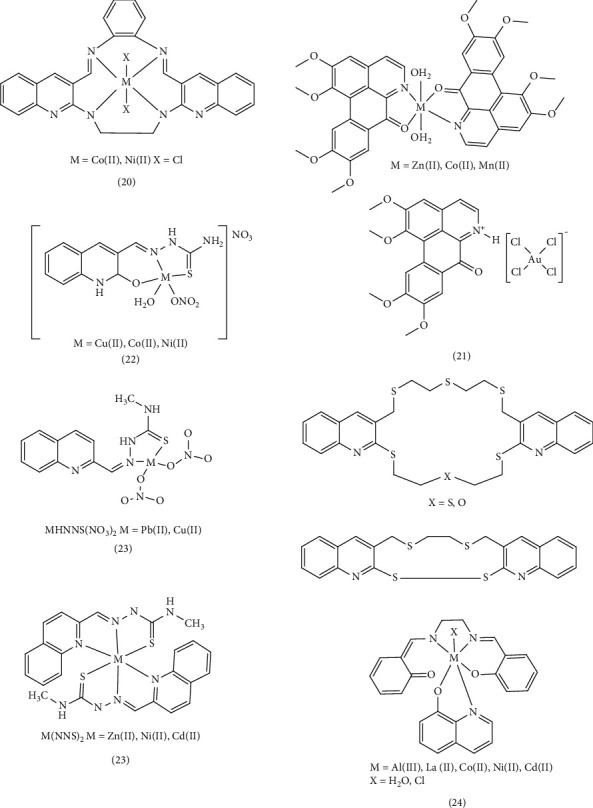
The chemical structure of quinoline metal complexes (20–24) with anticancer and antimicrobial activity.

In a follow-up investigation, the antibacterial activity of HNNS Schiff base and its corresponding Pb (II), Cu (II), Zn (II), Ni (II), and Cd (II) Complex 23 against Gram-positive and Gram-negative bacteria were assessed using the agar diffusion test. The Cu (NNS)NO_3_, Zn (NNS)_2_, and Cd (NNS)_2_ complexes (NNS = anionic form of the Schiff base of quinoline-2-carboxaldehyde 4-methyl-3-thiosemicarbazone) demonstrated more inhibition compared to the free HNNS ligand. While PbHNNS (NO_3_)_2_ showed only mild inhibition, no inhibition was observed for the Ni (NNS)_2_ complex. The research indicated that the complexes, particularly copper, zinc, and cadmium, exhibited higher efficacy against strains of *B. subtilis* compared to other strains, possibly due to their increased affinity for the bacteria [56]. Furthermore, the agar diffusion test demonstrated antibacterial and antifungal activity against Gram-positive and Gram-negative bacteria and two fungi. In terms of antibacterial efficacy, the order of potency was found to be AlSQ > LaSQ > CdSQ > gentamicin > NiSQ > CoSQ > Q > S for the MSQ metal complexes (Complexes 24) free salen (S), derived from (2,20- 1,2-ethanediylbis [nitrilo(E) methylylidene] diphenol, and 8-hydroxyquinoline (Q), as well as the standard drug (gentamicin). Similarly, antifungal activity exhibited the order of LaSQ > AlSQ > CdSQ > ketoconazole > NiSQ > CoSQ > Q > S. After the formation of the complex, the difference in the energy gap decreases and the electron transfers between the metal and the ligand increase. This results in enhanced lipophilicity of the complex, facilitating its penetration through the cell membrane and inhibiting cell proliferation. According to the calculations made in the article, the gap energy level difference in the lanthanum complex is less than other complexes, suggesting a higher antimicrobial property [75]. Muhammad et al. designed and synthesized new crown ethers that incorporate sulfur atoms based on quinoline and their complexes containing Cu^2+^, Cd^2+^, Hg^2+^, Zn^2+^, and Ag^+^. The conductometric titration method was employed to determine the stability constants. The stability constants for the metal cations followed the order of Hg^2+^ > Ag^+^ > Cu^2+^ > Zn^2+^. The higher the electrode potential of the standard reduction of the central atom (E^o^), the greater the tendency of the metal to accept an electron from the ligand, leading to a more stable complex. Cytotoxicity in these compounds decreases with increasing stability. Given the intriguing physiological and biological properties of quinolines and their derivatives, the biological activity of synthetic compounds was investigated. These compounds exhibited inactivity against both Gram-negative and Gram-positive bacteria concerning antioxidant and antibacterial activities, as determined by the agar diffusion test method. They also showed a weak cytotoxic effect on the MDA MB-231 cell line (human breast adenocarcinoma) and ATCC HTB-26 (breast tumor cells). It was hypothesized that crown compounds could be complexed with metal ions in prokaryotic or eukaryotic cells, potentially disrupting ion homeostasis or inhibiting the activity of some vital enzymes in these cells [57] (Figure 5).

**Figure 6:**
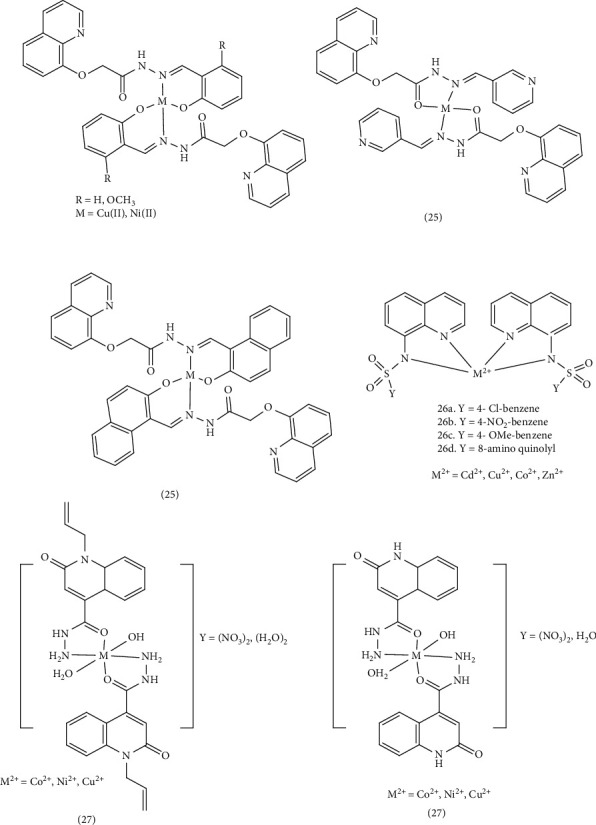
The structure of quinoline metal complexes (25–27) with antimicrobial activity.

Hanan et al. synthesized four Schiff base ligands (*N′*-[(*E*)-(2-hydroxyphenyl) methylidene]-2-[(quinolin-8-yl)oxy] acetohydrazide (SL_1_), *N*′-[(*E*)-(2-hydroxy-3- methoxyphenyl) methylidene]-2-[(quinolin-8-yl) oxy]acetohydrazide (SL_2_), *N*′-[(*E*)-(2-hydroxynaphthalen-1-yl) methylidene]-2-[(quinolin-8-yl)oxy] acetohydrazide (SL_3_)) with hydrazine quinoline scaffold and mononuclear Cu (II) and Zn (II) metal complexes, 25. The chelates were formed by connecting these ligands through the N atom of the azomethine group and the O atom of the phenolic or carbonyl group with metal ions Cu (II) and Zn (II). In vitro conditions, the antibacterial and antifungal potential of the synthesized compounds were investigated. The ligands exhibited moderate antibacterial activity against Gram-positive bacteria, while metal complexes showed significantly increased activity against these strains, albeit with a smaller inhibition zone (IZ) than the standard reference drug. But ligands and complexes were inactive against Gram-negative bacteria. In silico studies revealed high gastrointestinal (GI) absorption for ligands and low GI absorption for complexes. Ligands showed good bioavailability, while complexes exhibited moderate to poor bioavailability [58]. Designed and synthesized two new series of quinoline sulfonamide hybrid derivatives and (M^2+^: Cd^2+^, Cu^2+^, Co^2+^, and Zn^2+^) complexes. Complex 26a demonstrated the highest antibacterial and antifungal activity compared to other complexes. Based on the concepts of Orton's cell permeability and chelation theory, the increased lipophilicity of the complexes, attributed to ligand-to-metal charge transfers (LMCT) and π electrons transfer, facilitated penetration of the cell membrane and inhibited cell proliferation. The substitutions of chlorine, fluorine, and bromine in the para position of the phenyl ring do not have a significant effect on the strength of the intramolecular hydrogen bond, but they cause the displacement of the vibrational bands related to the phenyl ring and increase the electron transfer in the whole complex compared to other substitutions of other complexes. The Mueller–Hinton agar method revealed significant antibacterial activity against *S. aureus* strain and *E. coli*. In addition, the Sabouraud agar method showed antifungal activity with a diameter of 25 mm (MIC = 9.04 × 10^−5^ mg/mL) against *Candida albicans* [59]. Ibrahim et al. synthesized new derivatives of quinoline [2-oxo-1,2-dihydroquinoline-4-carbohydrazide, 2-(allyloxy) quinoline-4-carbohydrizde, 1-allyl-2-oxo-1,2-dihydroquinoline-4-carbohydrazid, and 2-(allylthio) quinoline-4-carbohydrazide] and their Cu (II), Ni (II), and Co (II) Complex 27. The synthetic compounds demonstrated significant activities against Gram-positive and Gram-negative bacteria and target fungi. Chelation increased the displacement of π electrons, enhancing the lipophilicity of the complexes. Smaller particle size facilitated penetration of microbial cell walls and cytoplasmic membranes, disrupting DNA and preventing reproduction. Density functional theory (DFT) and in silico studies, including molecular docking simulations with *E. coli* (PDB ID: 5C9T) were conducted to investigate the required properties and binding mechanisms. As a result of these investigations, the Cu (II) complex demonstrated the highest antimicrobial activity, exceeding even the approved drugs fluconazole and ampicillin, when compared to the Ni (II) and Co (II) complexes. Furthermore, the complexes demonstrated greater activity than their corresponding ligands [66] (Figure 6).

**Figure 7:**
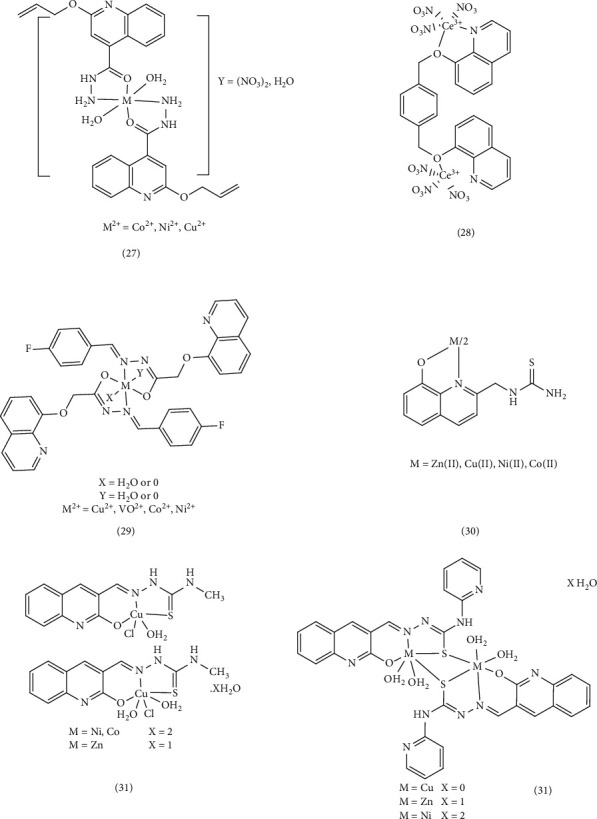
The structure of quinoline metal complexes (27–31) with antimicrobial activity.

Bhuvanesh et al. investigated quinoline complexes as fluorescent probes for live cell imaging to detect cerium ions. They observed that Ce^3+^ ions exhibited π to π^∗^ transition and intramolecular charge transfer (ICT) mechanisms during the recognition process. Utilizing a confocal laser scanning microscope, they assessed the performance of these probes in biological environments and evaluated their antibacterial activity against two pathogenic bacteria: *S. aureus,* and *E. coli*. In vitro cell survival was determined using the MTT method. The fluorescence emission observed in the cultured cells during imaging studies revealed the viability of the cells in light microscope images. Two Ce^3+^ Complex 28 demonstrated promising antifungal and antibacterial activity. The higher the reduction potential E^o^ of the central atom, the greater its tendency to accept electrons from the ligand leading to a more stable complex. The percentage of cellular inhibition was calculated, and IC_50_ values were reported as 43.32 ± 2.35 μM and 41.17 ± 2.15 μM [61]. El-saied et al. synthesized hydrazone Complex 29, incorporating M^2+^ ions such as Cu^2+^, VO^2+^, Co^2+^, Ni^2+^, and Zn^2+^ with quinoline-based ligands. These compounds were tested for their inhibitory effects against both Gram-positive and Gram-negative bacteria using the well diffusion method. The IZ was measured in millimeters, and the activity index (AI) values were determined. Based on the Overtone and Tweedy's theory of chelation, reducing the polarity of the central atom by chelating with a ligand increases the displacement of π electrons, thereby enhancing the lipophilicity of the complexes and facilitating their penetration into lipid membranes. Notably, the copper complex exhibited a more potent inhibitory effect compared to other complexes and hydrazone ligand alone [62]. Abuthahir et al. investigated the antibacterial activity and synthesis of metal complexes of a quinoline ligand (1-(8-hydroxy quinolin-2yl-methyl) thiourea ligand (HTF)) comprising Zn (II), Cu (II), Ni (II), and Co (II) Complex 30. The Cu (II) and Co (II) complexes demonstrated effective inhibition against both Gram-positive and negative bacteria, particularly against *S. aureus*, compared to the ligand. Furthermore, Zn (II), Cu (II), and Ni (II) metal complexes exhibited superior activity against *E. coli* bacteria. As previously mentioned, chelation and enhanced electron transfer between the metal and ligand increase lipophilicity and cell membrane penetration, thereby preventing bacterial proliferation [63]. In another study focused on the synthesis of quinoline–thiosemicarbazone derivatives and their Co (II), Ni (II), Cu (II), and Zn (II) Complex 31. An investigation in to antimicrobial properties these synthetic compounds revealed that copper and nickel complexes displayed greater activity compared to their corresponding ligands, particularly the copper complexes, which exhibited significant activity against *Aspergillus niger* compared to Gram-positive and Gram-negative bacteria. The ligand-drug complex regulates lipophilicity, prevents enzymatic degradation, and enhances both inhibitory effect and antimicrobial activity [64]. (Figure 7).

**Figure 8:**
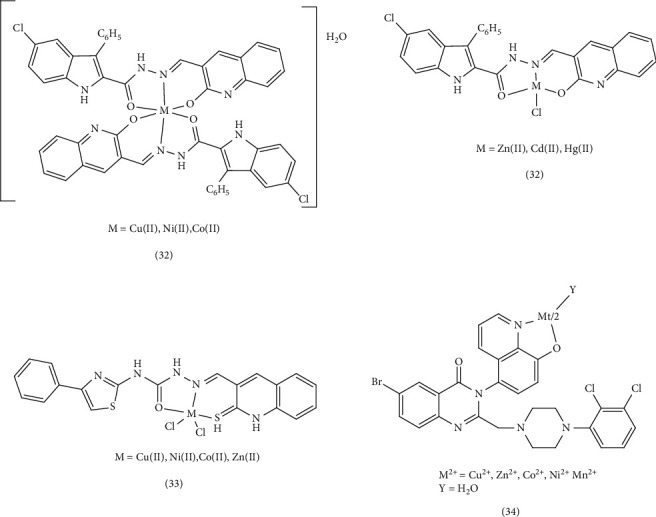
The structure of quinoline metal complexes (32–34) with antimicrobial activity.

In another study, new complexes of Cu (II), Co (II), Ni (II), Cd (II), and Hg (II) (Complex 32) were designed and synthesized using quinoline and indole derivatives. These derivatives were based on the Schiff base of 5-chloro-3-phenyl-1H-indole-2-carboxyhydrazide and 3-formyl-2-hydroxy-1H-quinoline (HL). The antimicrobial activities of the synthesized compounds were evaluated using the minimum inhibitory concentration (MIC) method against *E. coli, S. typhi, B. subtilis, S. aureus, C. albicans, C. oxysporum, and A. niger*. In addition, the DNA cleavage ability of the compounds was assessed using agarose gel electrophoresis with *E. coli* DNA. The MIC values indicated that the heterocyclic Schiff-base complexes effectively penetrated the bacterial membrane lipid layer due to the displacement of the *π* electron within the entire chelate, leading to increasing lipophilicity of the metal chelate. DNA cutting studies revealed that the Cu (II) complex completely cleaved the genomic DNA of *E. coli*. This activity can be attributed to the formation of nitrogen and oxygen chelation with the metal, which inhibits the growth of pathogenic organisms [65]. Yernale et al. conducted a study on the synthesis and in vitro cytotoxicity studies of Schiff-base ligand derived from thiazole and quinoline (N-(4-phenylthiazol-2yl)-2-((2-thiaxo-1,2-dihydroquinolin-3-yl) methylene) hydrazinecarboxamide (L)), as well as Cu (II), Co (II), Ni (II), and Zn (II) Complex 33 of the ligand. These metal complexes were tested in vitro for their antimicrobial activity against *Enterobacter aerogenes*, *Pseudomonas aeruginosa*, *A. niger*, and *A. flavus* fungal strains. The metal complexes exhibited superior antimicrobial activity compared to the free ligand, which can be attributed to electron displacements and increased lipophilicity of the metal chelates [66]. Vashi et al. studied the synthesis and antifungal activity of quinoline ligands (6-bromo-2[(4-(2,3-dichlorophenyl)) piperazin-1-yl) methyl]-3-[8-hydroxyquinolin-5-yl]- 3(H)-quinazolin-4-one (HL)) and metal chelates (Complex 34). They tested these compounds against various fungi, including *C. albicans*, *Botrydepladia thibromine*, Nigrospora Sp, *A. fumigatus*, and *Rhizopur nigricums*. The Schiff-base ligand displayed the highest antifungal activity compared to the Co (II), Cu (II), Zn (II), Mn (II), and Ni (II) complexes. Substituting the phenyl rings with chlorine, increased antifungal activity, suggesting these compounds could potentially serve as antifungals against specific plant pathogenic organisms [67] (Figure 8).

**Figure 9:**
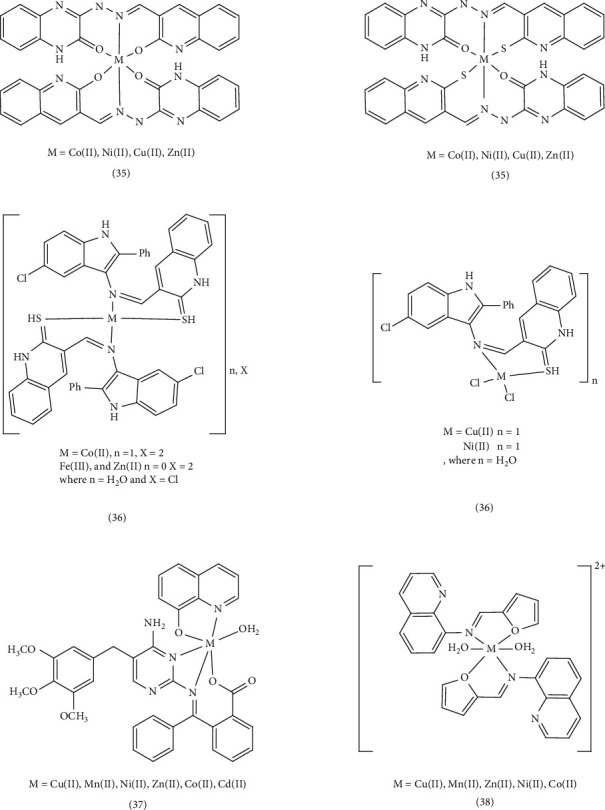
The structure of quinoline metal complexes (35–38) with antimicrobial activity.

Nath et al. also synthesized Schiff bases (3-quinoxalin-2-ol (QZOH) and 3-(2E)-2-[(2-sulfanylquinolin-3-yl) methylidene]hydrazinyl)quinoxalin-2-ol (QZSH)) and their Co (II), Ni (II), Cu (II), and Zn (II) Complex 35 and evaluated their antibacterial and antifungal properties under in vitro conditions. The antibacterial and antifungal activities of the synthesized ligands and their metal complexes were assessed using the potato dextrose agar diffusion method against *E. coli* and *S. aureus* bacteria, as well as *A. niger* and *P. chrysogenum* fungi. These results were then compared to those obtained from standard antibacterial (gentamicin) and antifungal (fluconazole) drugs. The assay results indicated that the smaller the central metal atom, the easier it is to penetrate the cell membrane. Copper ions are smaller than other ions, as a result, the Cu (II) complex exhibited greater bactericidal activity than other complexes [68]. Vivekanand et al. evaluated the antimicrobial, DNA-cutting, and antioxidant properties of the Schiff-base quinoline (5-chloro-2-phenyl-1H-indol-3-ylimino)methyl)quinoline-2(1H)-thione) and its metal Complexes 36. The cup-plate method with nutrient agar media was used to assess the bactericidal efficacy of the ligand and its metal complexes. During these experiments, Cu (II), Co (II), and Fe (III) complexes were more effective against *S. aureus* and *P. aeruginosa* than the other complexes. Using the cellulose dextrose agar (SDA) method to evaluate antifungal activity, Cu (II) and Co (II) complexes were more effective against *A. niger* and *A. flavus* than their synthetic counterparts. The function and effect of metal complexes depend on the nature of the metal ions and their electrochemical properties. Complexes with a lower reduction potential showed more biological activity, leading to the acceleration of drug action and an increase in their therapeutic properties [69]. Numan et al. also discussed synthesizing and studying antibacterial Schiff-base ligands (2-((4-amino-5-(3, 4, 5-trimethoxybenzyl) pyrimidin-2-ylimino)(phenyl)methybenzoic acid) [HL]) and their Complex 37. Using the nutrient agar diffusion method, in vitro antibacterial activities of ligands and their complexes against *S. aureus*, *E. coli*, *E. cloacae*, and *B. subtilis* were tested. Based on the obtained results, they discovered the antibacterial activity of the Schiff-base ligand and its zinc and copper complexes. Based on the electron transfers stated in the article, with the formation of metal complexes and the increase of these transfers, the lipophilicity and penetration into the cell membrane increases, leading to prevention of reproduction, turning off enzymes, and disrupting the metabolism of microbial cells [70]. Shakir et al. reviewed the synthesis and cytotoxic, antibacterial, and antioxidant studies of pharmacologically important Mn (II), Co (II), Ni (II), Cu (II), and Zn (II) Complex 38 of the quinoline Schiff-base ligand ((E)-N-(furan-2-ylmethylene)quinolin-8-amine). The Schiff-base ligand and its metal complexes were tested against ampicillin and amphotericin B on three Gram-negative bacteria, two Gram-positive bacteria, and two yeasts (*C. albicans*, MTCC-227 and *A. niger*, MTCC-1881). The Mn (II) and Co (II) combination had bacterial activity comparable to ampicillin (MIC: 31.5 μg/mL). The complexes' activity level depends on the type of cellular ribosomes and the level of impermeability of microbial cells. Increasing the solubility of lipids in the complexes enables metal ions to achieve optimal cell wall function and halt cell proliferation. In addition to chelation, the geometry of the complexes, the concentration and potential of oxidation, and reduction of metal ions, as well as numerous other factors can significantly affect the antimicrobial activity of complexes [80] (Figure 9).

**Figure 10:**
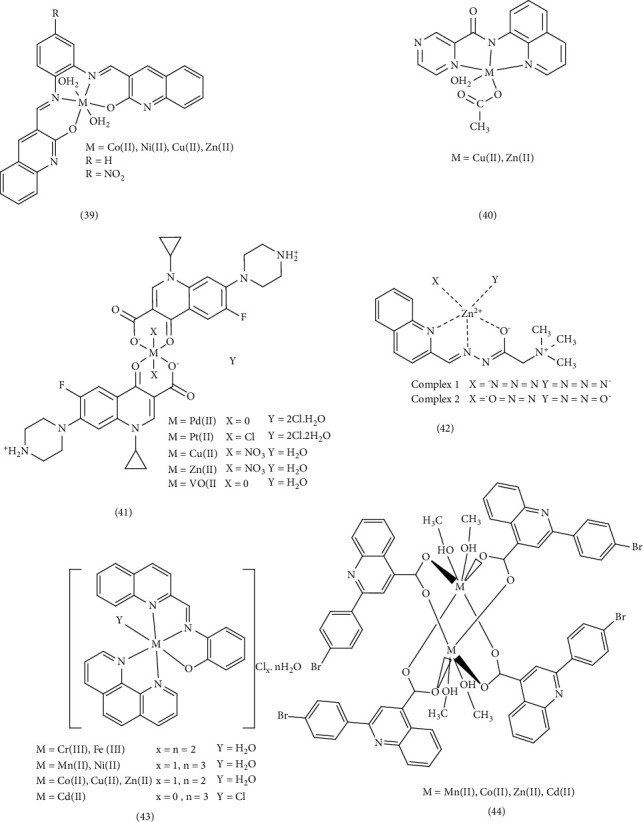
The structure of quinoline metal complexes (39–44) with antimicrobial activity.

Narayanachar et al. synthesized novel quinoline ligands (L1 and L2), formed by reacting 2-oxo-3-quinoline-3-carbaldehyde with substituted *ortho*-phenylinediamine (*o*-phen and nitro-*o*-phen), respectively. They then investigated the microbial activity of the corresponding Co (II), Ni (II), Cu (II), and Zn (II) Complexes 39. The Cu (II) complexes, [Cu(L1) (H_2_O)_2_] and [Cu(L2) (H_2_O) _2_] exhibited potent inhibitory effects. The observed shifts in electronic absorption bands and electronic transfer within the complexes indicated chelation, which increased their lipophilicity. This enhanced lipophilicity, coupled with the smaller atomic size of Cu (II), facilitated greater the penetration in to microbial cells, leading to increased antimicrobial activity [71]. Meghdadi et al. employed the ionic liquid tetrabutylammonium bromide (TBAB) to synthesize a new quinoline-derived ligand and its two Zn (II) and Cu (II) Complex 40. Their antibacterial activity was evaluated against Gram-positive *S. aureus* and Gram-negative *E. coli* using Sabouraud dextrose agar and Müller–Hinton agar methods. The Cu (II) complex demonstrated stronger antibacterial activity compared to the Zn (II) complex, its ligand, and the standard antibiotic penicillin. This enhanced activity was attributed to the stronger Cu (II)-ligand bond, which promoted increased lipophilicity, improved permeability across the cell membrane, and protection against enzymatic degradation [72]. Gamil et al. explored the antibiotic activity of a series of quinoline drug complexes, including ciprofloxacin (1-cyclopropyl–6–fluoro–4–oxo-7-(piperazin–1-yl)-1,4-dihydroquinoline-3-carboxylic acid (ciproH)). Using agar disk diffusion tests, they evaluated the antimicrobial activities of this Complex 41, against various Gram-positive (Bacillus sp., Klebsiella sp., and Proteus sp.) and Gram-negative (Klebsiella sp. and Proteus sp.) microorganisms. The majority of the complexes exhibited sensitivity to these microorganisms, and some displaying bacteriostatic activity. Certain complexes even outperformed the standard antibiotic drug (ciproH). The variations in the inhibitory effects were likely due to the differences in the penetration of the compounds through the cell membrane [73]. Ronaovic et al. synthesized zinc (II) Complex 42 and evaluated their antimicrobial activity against Gram-positive and Gram-negative bacteria, the yeast *C. albicans*. The synthetic ligand (L), formed by the reaction of 2-quinolinecarboxaldehyde and trimethylammonium acetohydrazide chloride (Girard's reagent T), displayed significant antimicrobial activity against *E. faecalis*. However, the antimicrobial activity increased upon the formation of [ZnL(N_3_)_2_] and [ZnL (NCO)_2_] complexes due to enhanced lipophilicity, stability, steric, and electronic properties. As a result, these zinc complexes exhibited the highest antimicrobial activity against *B. subtilis* and *K. pneumoniae*. The dinuclear azido bridged Ni (II) complex showed limited activity against *C. albicans* (MIC value of 0.571 mM) but was less potent than standard drugs [74]. El-Halim et al. synthesized a quinoline Schiff-base ligand (HL) form quinoline‐2‐carboxaldhyde and 2‐aminophenol, along with its Mn (II), Fe (III), Ni (II), and Cu (II) Complex 43. The antibacterial activity of these complexes revealed that they exhibited relatively lower activity against the Gram-positive bacterium *S. pneumoniae* compared to the Schiff-base ligand. However, the Cr (III), Co (II), Ni (II), Cu (II), Zn (II), and Cd (II) complexes were more effective than the Schiff-base ligand against *B. subtilis*. Conversely, the Cr (III) and Fe (III) complexes were less effective than the Schiff-base ligand against the Gram-negative bacterium *P. aeruginosa*. Compared to the Schiff-base ligand, the activity of Cr (III), Fe (III), and Ni (II) complexes against *E. coli* was weaker. When tested for antifungal activity, all of the compounds except for the Cr (III) complex and the Ni (II) complex, outperformed the Schiff-base ligand. This enhanced activity could be attributed to the presence of chlorine, which may combine with membrane proteins and enzymes to boost its antibacterial effectiveness. Alternatively, the formation of HCl and O_2_ within the complex could lead to oxidation of the cell. The lower activity of metal complexes such as Cr (III) and Ni (II) might be due to their ability to decrease lipid fluidity by reducing π-electron movement, thereby hindering penetration into the lipid membrane [87, 88]. Qin et al. investigated the synthesis and antibacterial evaluation of metal complexes of 2-phenylquinoline derivatives (Complex 44). Compared to reference antibiotics streptomycin, penicillin, and ciprofloxacin, these complexes exhibited better antibacterial activity than the free ligands or metal salts alone. Notably, the Zn (II) and Cd (II) complexes demonstrated high antibacterial activity against *S. aureus* with IC_50_ = 0.57 μg/mL and 0.51 μg/mL, respectively, surpassing the positive controls [75]. (Figure 10).

**Figure 11:**
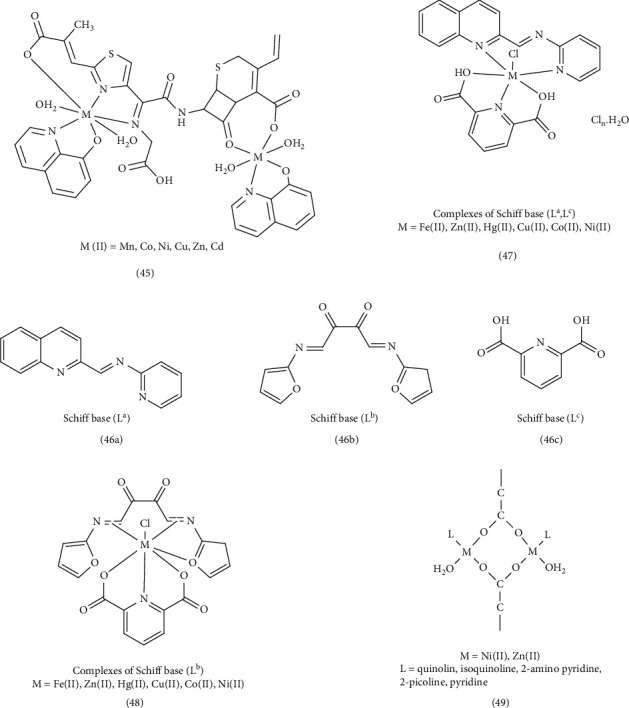
The structure of quinoline metal complexes (45–49) with antimicrobial activity.

In the study by Numan et al., the biological activities of several Complex 45 were investigated. These complexes included 8-hydroxy quinoline, a Schiff-base ligand (7-((E)-2-(2-((Z)-1- carboxylatoethylid eneamino) thiazol-4-yl)-2-(carboxylatomethoxyimino) acetamido)-8-oxo-3-vinyl-5-thia-1- azabi-cyclo[4.2.0]oct-2-ene-2- carboxylate (L)), and transition metal ions (Mn, Co, Ni, Cu, Zn, and Cd). The results indicated that all the complexes exhibited significant antibacterial activity against bacteria. However, the [(Ni)2(Q)2(KL) (H2O)4] complex did not demonstrate inhibitory activity against *E coli*. Similarly, the 8-hydroxy quinoline ligand showed no effect on pseudomonas. Conversely, the cefixime Schiff-base ligand (NaK2L) exhibited anti-Staphylococcus activity [89]. Hanan et al. examined the characteristics and reactivity of two quinoline Schiff-base ligands (46a and 46b) and their complexes with Fe (II), Co (II), Ni (II), Cu (II), Hg (II), Ni (II), and Zn (II), along with a secondary ligand (46c). Ligand 46a was synthesized by mixing quinoline 2‐aldehyde with 2‐aminopyridine, while ligand 46b was synthesized by mixing oxamide with furfural. Ligand 46c was 2,6-pyridinedicarboxylic acid. This study aimed to evaluate the potential of these complexes as antibacterial and anticancer agents, as reported in a previous publication. Agar diffusion method results revealed that among the synthesized ligands and complexes tested, Ligand 46c exhibited the most potent antibacterial activity against *S. pneumonia*, *B. subtilis*, *P. aeruginosa*, and *E. coli*. Antifungal activity against *A. fumigates* was observed with the Ni (II) and Co (II) complexes of Ligand 46c, (Complex 47) and antifungal activity against *C. albicans* with the Ni (II), Co (II), and Fe (II) complexes of Ligand 46b (Complex 48). The authors concluded that the enhanced antimicrobial activity might be attributed to the chelation process, where the positive charge of the metal is shared with the donor groups. This chelation increases the lipophilic nature of the central atom, facilitating penetration into the cell membrane. The smaller the central atom, the stronger the bond (M-O) and (M-N) bonds, further enhancing the rate of penetration and antimicrobial activity. The molecular docking simulation investigations of ligands and complexes against receptors of *E. coli* (PDB ID: 3t88) and mutant breast cancer oxidoreductase (PDB ID: 3hb5) suggested that the Zn (II) complex, which forms hydrogen and cation-π bonds, exhibits superior performance compared to all other synthesized compounds [89]. Khatun et al. synthesized and examined the antibacterial properties of nickel (II) and zinc (II) Complex 49. These complexes were formed with ligands containing aromatic rings, specifically L = quinoline, 2-picoline, pyridine, isoquinoline, 2-aminopyridine, α-pic = α-picoline, Py = pyridine. The antibacterial activity of the complexes, particularly the [Zn (DiPhAc) (L) (H_2_O)] _2_(NO_3_)_2_ complex, which included diphenyl acetic acid (DiPhAc) was evaluated against both Gram-positive and Gram-negative bacteria. Compared to the standard kanamycin, these complexes exhibited greater inhibitory potency and sensitivity. This enhanced activity was attributed to the specific characteristics of the metal ion, ligand, and their arrangement around the metal ion. Furthermore, the complexes demonstrated superior efficacy compared to the individual ligands and even surpassed the standard drug [77]. (Figure 11).

Patel et al. synthesized new quinoline ligand complexes containing Mn^2+^, Fe^2+^, Co^2+^, Ni^2+^, and Cu^2+^ Complex 50, using the ligand 5-((4-(6,7-dihydrothieno [3,2-c] pyridin-5(4H)-ylsulfonyl) phenylamino) methyl) quinolin-8-ol (HTPSMQol). They then screened these complexes for antimicrobial activity against various microorganisms. The results showed that the quinoline ligand itself exhibited superior and statistically significant antibacterial efficacy compared to both the metal complexes and the antibiotic ciprofloxacin. Complexes containing Fe (II) and Cu (II) exhibited best activity among the metal complexes. A separate study by other researchers synthesized a series of metal Complex 51, containing Cd (II), Cu (II), and Zn (II) with a quinoline Schiff-base ligand (3-[(E)-(4H-1,2,4-triazol-4-ylimino) methyl] quinoline-2-thiol (L)). The researchers investigated the interaction of these complexes with CT-DNA (calf thymus–deoxyribonucleic acid). The study found that the metal complexes, particularly the Cu (II) complexes, displayed higher antimicrobial activity compared to the free ligand against both Gram-positive and Gram-negative bacteria as well as two yeasts (*A. niger* and *C. albicans*). This enhanced activity is attributed to the smaller size of the copper atom, the strength of its bond with the ligand's donor atoms, and its greater ability to penetrate the cell membrane. In addition, the octahedral structure of the complexes is believed to play a significant role in their antimicrobial activity [90]. Zhang et al. synthesized four quinoline-based metal Complex 52, containing Mn (II), Co (II), Cd (II), and Ag (I), and compared their fluorescence and antibacterial properties to streptomycin. The Cd (II) complex showed the highest antibacterial activity against Gram-positive strains. Co (II), Cd (II) complexes were more effective against Gram-positive bacteria than Gram-negative bacteria. The Ag (I) complex displayed selective inhibition of the Gram-negative bacterium *P. aeruginosa* with an IC_50_ = 6.74 μg/mL. The study concluded that the geometry and central atom of the complex play crucial roles in their antibacterial activity. Complexes with higher standard reduction potentials (such as Cd (II) and Ag(I)) tend to form more stable complexes by accepting electrons from the ligand. This increased stability strengthens the bond with the ligand's donor atoms, facilitates electron transfer, and increases lipophilicity. As a result, these complexes can more easily penetrate cell membranes and inhibit cell proliferation [79]. Lamani et al. synthesized quinoline Complex 53 and investigated their anticancer, antifungal, and DNA binding capabilities in antimicrobial activity experiments. They found that the metal complexes exhibited greater inhibitory activity than the synthetic ligand (diquinolineno [1, 3, 7, 9] tetraazacyclododecine-7,15-ethane(14H, 16H)-benzene (L)), even surpassing the activity of chloramphenicol and fluconazole standards [85]. Hessah et al. explored a straightforward approach for generating a new set of Complex 54, containing Zn (II), Pd (II), VO (II), and Cr (III), derived from 4-bromo-2-(quinolin-8-yliminomethyl) (BSQ). The study found that the BSQPd complex displayed the strongest inhibition among all tested compounds [40]. Camila et al. successfully produced a series of fluorescent quinoline complexes containing Zn (II), Al (III), Cu (II), and Ru (II). These complexes exhibited both biological activity and fluorescence. The study observed low cytotoxicity towards blood cells and moderate antimicrobial effects. Complex 55 displayed enhanced efficacy against both Gram-positive and Gram-negative bacteria. This was attributed to the higher standard reduction electrode potential of Pd (II), which contributes to the formation of a more stable complex. By increasing the stability of the complexes, the electron transfer increases and the lipophilicity and penetration power to the cell membrane increases, and as a result, the antimicrobial activity increases [78].

### 3.3. Anthelmintic Activity

**Figure 12:**
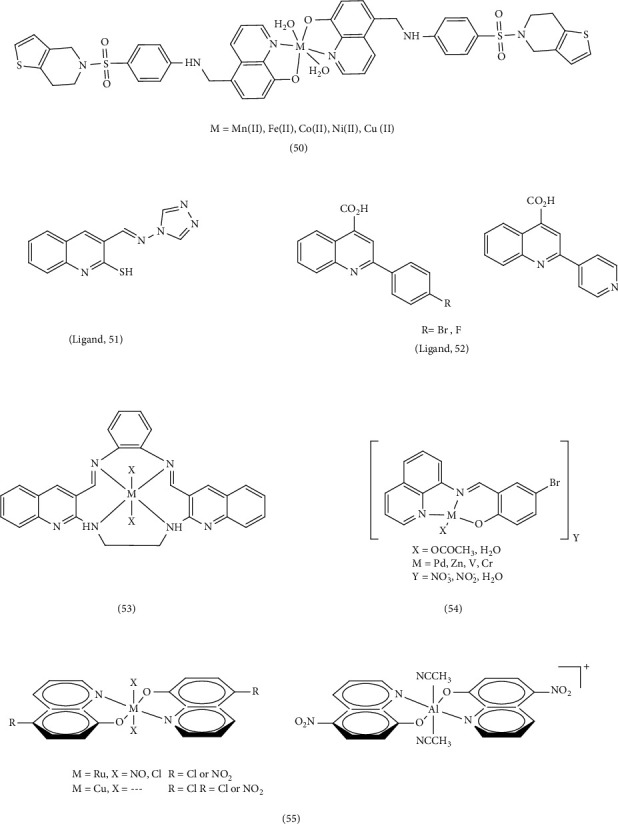
The structure of quinoline metal complexes (50–55) indicating anthelmintic and antimicrobial activity.

Lokesh et al. synthesized a series of metal Complex 51, M = Cd (II), Cu (II), and Zn (II) using a quinoline Schiff-base ligand, (3-[(E)-(4H-1, 2, 4-triazol-4-ylimino) methyl] quinoline-2- thiol (L). They investigated and evaluated the interaction of the complexes with CT-DNA (calf thymus–deoxyribonucleic acid). In laboratory conditions, the anthelmintic activity of the synthetic compounds was evaluated on earthworms (*Pheretima posthuma*). Compared to the standard drug albendazole, the Cu (II) and Zn (II) complexes demonstrated significant activity, resulting in complete paralysis and death of the worms. This activity may be attributed to their interference with the neuromuscular physiology of the parasites. It is also possible that the complexes inhibit the energy metabolism of the parasites by absorbing glucose and depleting of glycogen stores [90] (Figure 12).

### 3.4. DNA Cleavage Studies

**Figure 13:**
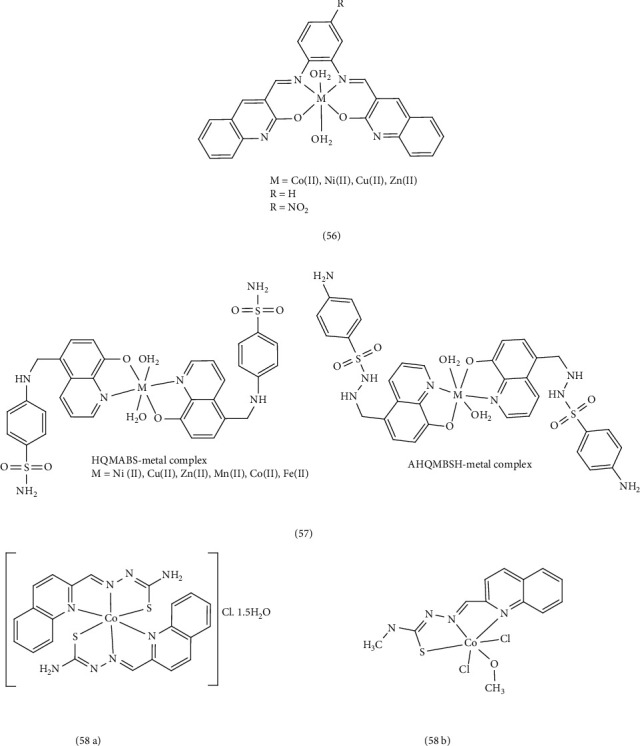
The structure of quinoline metal complexes (56–58) indicating DNA cleavage studies.

The investigation conducted by Narayanachar et al. focused on the examination of Co (II), Ni (II), Cu (II), and Zn (II) Complex 56 derived from novel quinoline ligands (a mixture of 2-oxo-3-quinoline-3-carbaldehyde and substituted *ortho*-phenylinediamine (*o*-phen, nitro-*o*-phen). They investigated the potential of these complexes to cleave DNA. Using agarose gel electrophoresis on *E. coli*, the researchers found that Co (II) and Ni (II) complexes exhibited DNA cleavage activity, leading to growth inhibition of the pathogenic organism. This finding suggests a significant role for metal ions in the cleavage reaction of isolated DNA. The research indicates that transition metal complexes can bind to DNA through both covalent and noncovalent interactions. Covalent binding occurs when the ligands of the complex are replaced by a portion of the DNA molecule, preventing DNA replication. Noncovalent binding can involve intercalation of aromatic heterocyclic rings between base pairs, binding to the major or minor grooves of DNA via hydrogen bonding and van der Waal interactions, or weak electrostatic binding to the DNA surface [71]. Dixit et al. designed and synthesized 8-hydroxyquinoline sulfonamide derivatives and their corresponding metal Complex 57. They evaluated the interaction of these complexes with plasmid DNA (pUC 19) and calf thymus DNA using gel electrophoresis and UV spectroscopy. DNA cleavage data revealed that Ni (II), Cu (II), and Zn (II) complexes exhibited the most potent cleavage activity, directly related to the ability of the ligands/complexes to bind DNA. Notably, complexes, particularly the Cu-AHQMBSH complex, displayed significantly stronger DNA binding affinity than the corresponding ligands. The AHQMBSH complexes exhibited a higher affinity for DNA binding compared to the HQMABS complexes, indicating a greater influence of metal ion-DNA interactions. In the octahedral structure, the copper ion can transition from a regular octahedral state to tetragonal arrangement for greater stability, through ion-teleomeric twisting. This twisting allows the copper ion to interact with DNA. Complexes with larger ligands, such as AHQMBSH, facilitate this twisting process [83]. Xiaorui et al. investigated the DNA interactions and cytotoxicity of two thiosemicarbazone Complex 58 of cobalt (II). Electronic absorption, circular dichroism (CD), and fluorescence spectroscopic analyses revealed that the Co (II) complex interacts strongly with DNA through groove binding, leading to DNA cleavage. Complex 58b exhibited a stronger DNA binding affinity compared to Complex 58a. The metal-ligand bond in Complex 58a is stronger, hindering its interaction with DNA. Conversely, the loosely connected ligands in Complex 58b facilitate a stronger interaction with DNA [82](Figure 13).

**Figure 14:**
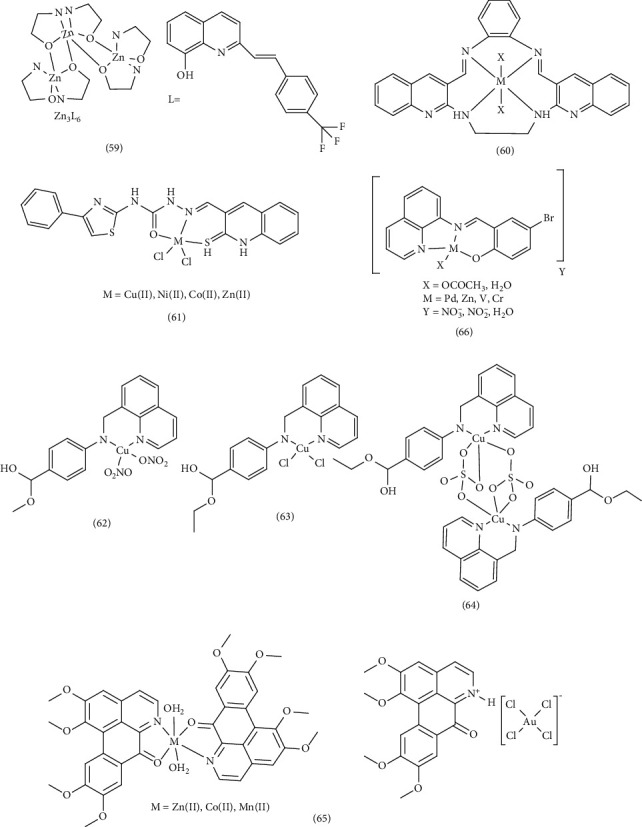
The structure of quinoline metal complexes (59–66) indicating DNA cleavage studies.

Using the solvothermal method, Yanping et al. successfully synthesized a novel trimeric complex of Zn (II) (Complex 59) based on quinoline specifically ((*E*)-2-[2-(4-trifluoromethylphenyl) ethenyl]-8-hydroxyquinoline (HL)). After analyzing its photophysical properties, they proceeded to investigate its binding mechanism with DNA. Adding the complex to DNA revealed changes in the EtBr-DNA system and the quenching rate constant K, confirming a strong interaction between the complex and DNA [84]. Following the synthesis of quinoline Complex 60 under laboratory conditions, Lamani et al. investigated their anticancer properties, antifungal activities, and binding affinity toward DNA. Employing absorption methodologies, emission spectroscopy, viscosity analysis, and thermal measurements, the binding kinetics of these complexes with calf thymus DNA were explored. The bathochromic and hypochromic alterations in the absorption spectrum, coupled with the assessment of the intrinsic binding constant, demonstrated a higher affinity of the Co (II) complex toward CT-DNA compared to the Ni (II) complex. Furthermore, the presence of these complexes led to an increase in the viscosity of DNA fragments [85]. Yernale et al. conducted an investigation into the synthesis and in vitro cytotoxicity evaluations of a Schiff-base ligand (N-(4-phenylthiazol-2yl)-2-((2-thiaxo-1,2-dihydroquinolin-3-yl) methylene) hydrazinecarboxamide (L)) derived from thiazole and quinoline, along with its corresponding complexes involving copper (II), cobalt (II), nickel (II), and zinc (II) (Complexes 61). During the analysis of DNA cleavage using agarose gel electrophoresis, the researchers explored the interaction between plasmid pBR322 DNA and synthetic compounds. They observed alterations in band patterns, indicative of the influence of metal complexes and ligands on DNA. In the in vitro cytotoxicity assessments conducted on saltwater shrimp utilizing the Meyer method, a comparison with *Artemia salina* was made. The findings indicated that the complexes exhibited superior biological activities compared to the ligands. In an independent research study, Kun et al. meticulously designed and synthesized three distinct copper complexes, denoted as [Cu(L1) (NO_3_)_2_] (Complex 62), [Cu(L2) Cl_2_] (Complex 63), and [Cu(L2) SO_4_]_2_H_2_O (Complex 64). These complexes were derived from Schiff-based ligands incorporating the quinoline moiety (quinoline-8-carbaldehyde with 4-amino-benzoic acid methyl ester or 4-amino-benzoic acid ethyl ester (benzocaine)). The precise interactions of Complexes 62–64 with HSA and calf thymus DNA (CT-DNA) were investigated using spectroscopic techniques and molecular docking analysis. The study presents the optimal binding positions identified through molecular docking of Complexes 62–64 with B-DNA (PDB ID: 1BNA) and HSA. Notably, Complexes 62–64 exhibited the lowest energies in these binding studies. Concurrently, Complexes 62–64 were strategically positioned within the active site of B-DNA, specifically within the minor groove region. This positioning was facilitated by a combination of hydrogen bonds, hydrophobic interactions, and van der Waals forces with functional groups present in the DNA structure. Furthermore, it was observed that their binding affinity with HSA surpassed that of other copper (II) complexes [73]. Chen et al. achieved the successful synthesis of OG along with four complexes denoted as (Complex 65) [M (OG)_2_(H_2_O)_2_] (ClO_4_)_2_)], where M represents Mn (II), Co (II), Zn (II), and Au (III). Applying techniques such as agarose gel electrophoresis assay, UV-vis spectroscopy, fluorescence analysis, CD spectroscopy, and viscosity measurements, researchers investigated the interactions of these metal complexes with calf thymus DNA (CT-DNA). These interactions primarily involved intercalation through both covalent and noncovalent binding mechanisms, including internalization, insertion, groove binding, and electrostatic attachment. These intricated interactions ultimately leading to the inhibition of topoisomerase I (TOPO I) [86]. In another study, Hessah et al. developed a straightforward method to synthesize a new series of complexes involving Zn (II), Pd (II), VO (II), and Cr (III) (Complex 66). These complexes were derived from the ligand BSQ (4-bromo-2-quinolin-8-yliminomethyl). The analysis included spectrophotometric titration and hydrodynamic techniques to explore their interactions with DNA. The results revealed that changes in absorption bands and DNA viscosity serve as clear indicators of the interactions between the complexes and DNA [40] (Figure 14).

### 3.5. Photodynamic Activity

Jia et al. synthesized several Zn (II) phthalocyanines derived from quinoline (oligomeric ethylene glycol−quinoline) and investigated their photodynamic activity in vitro (Complex 67). They assessed the photodynamic activities by examining the cytotoxic effects of these substances on human HepG2 liver cells using confocal microscopy. Monosubstituted phthalocyanines exhibited high phototoxicity to HepG2 cells with IC_50_ values of 0.02–0.05 M in the presence of light. This high photodynamic activity is attributed to their low accumulation and high cellular uptake, allowing them to target mitochondria and lysosomes. Tetrasubstituted phthalocyanines, on the other hand, primarily affect lysosomes. This suggests that monosubstituted phthalocyanines hold potential as highly effective antitumor agents for photodynamic therapy [80].

### 3.6. Antitubercular Activity

Mandewale et al. synthesized and examined the antituberculosis activity of quinoline-based hydrazone derivatives and their copper (II) and zinc (II) Complex 68 against Mycobacterium tuberculosis strain (H37 RV). The metal complexes demonstrated greater efficacy than free hydrazone ligands, potentially due to the transfer of electrons from the ligand to the metal, enhancing lipophilicity and facilitating penetration through the bacteria's lipid cell membrane. This improved permeability enables the complexes to disrupt cellular respiration, inhibit cell wall formation, and ultimately induce cell death. In addition, the metal complexes may inhibit cell growth by interfering with the function of the DNA gyrase enzyme. The inclusion of a heterocyclic ring, such as a quinoline moiety, within the molecular structure of hydrazone in synthetic complexes has been observed to significantly impact the growth inhibition of Mycobacterium tuberculosis [35].

**Figure 15:**
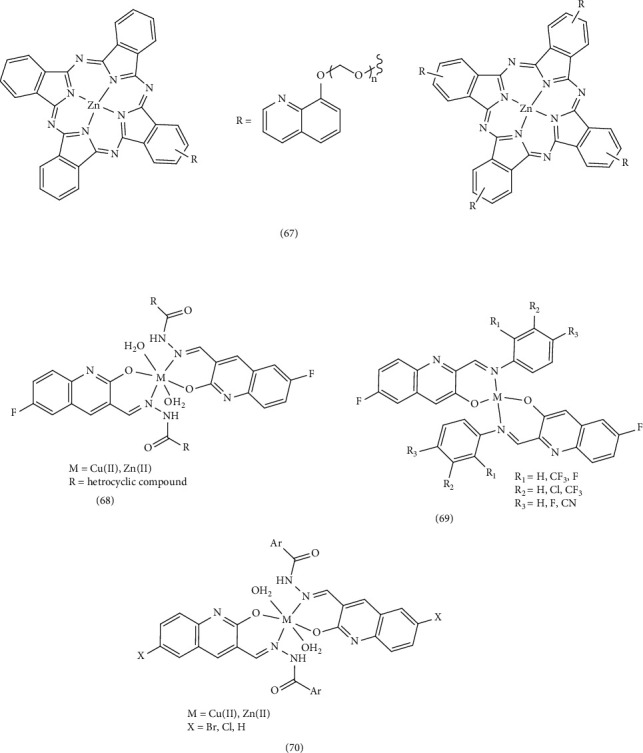
The structure of quinoline metal complexes (67–70) with photodynamic and antitubercular activity.

In a separate study, Mandewale et al. synthesized five quinoline Schiff bases (a mixture of 6-fluoro-2-hydroxyquinoline-3-carbaldehyde and substituted aniline) and assessed the antituberculosis activity of their copper (II) and zinc (II) Complex 69. Using the nontoxic Alamar Blue (MABA) microplate method, they found that zinc (II) complexes exhibited superior antibacterial efficacy against *Mycobacterium tuberculosis* (H37 RV strain) compared to copper (II) complexes. Further analysis revealed that zinc (II) complexes possess stronger fluorescent properties than copper (II) complexes [91]. In 2016, Mandewale et al. synthesized and investigated a range of hydrazone derivatives based on quinoline, along with their copper (II) and zinc (II) Complex 70. Utilizing Blue Alamar technique, fluorescence, and ultraviolet (UV) experiments, they evaluated the compounds' antituberculosis activity. The results indicated that a majority of the synthesized compounds, particularly the zinc complexes, exhibited favorable antituberculosis activity, particularly the complexes surpassing the efficacy of the reference compounds ciprofloxacin and streptomycin. Complexes, where *X* represents Cl, Br, and Ar represents pyridine, have shown significant ability to infiltrate bacterial cells. However, the effectiveness of these compounds against M. tuberculosis has shown to be unpredictable [13] (Figure 15).

### 3.7. Neuroprotective Studies

**Figure 16:**
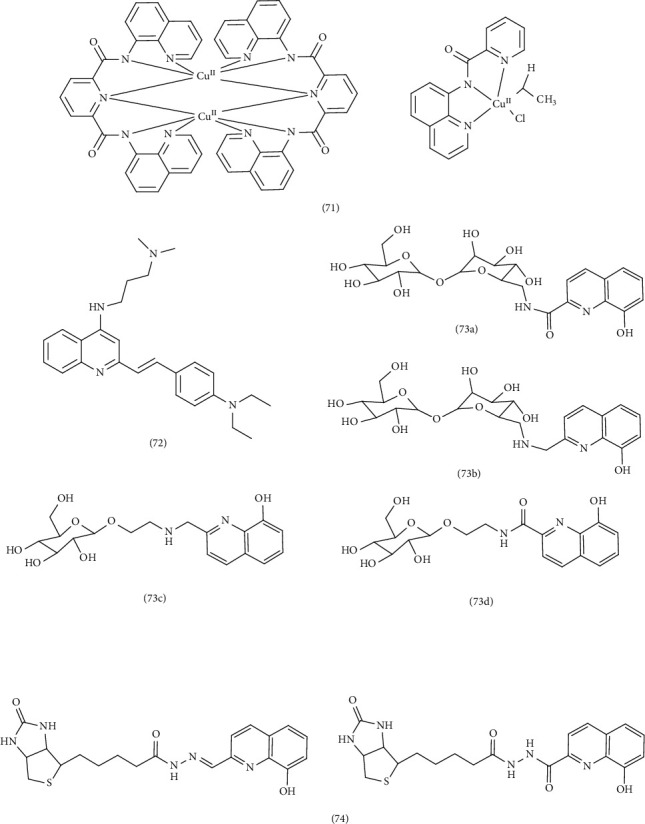
The structure of quinoline metal complexes (71–74) with neuroprotective studies.

Nikolaos et al. synthesized ligands featuring a quinoline scaffold: *N*-(8-quinolyl) pyridine-2-carboxamide (Hbpq), *N*^2^, *N*^6^-di(quinolin-8-yl) pyridine-2,6-dicarboxamide (H_2_dqpyca), and *N,N*′*-*Bis(8-quinolyl) cyclohexane-1,2-diamine (H_2_bqch), along with their corresponding Cu (II) Complex 71. These compounds were investigated for their neuroprotective properties in the context of Alzheimer's drug development. And a thorough investigation of their physical and chemical properties was conducted. All of these compounds were subjected to pharmacological evaluation, revealing notable outcomes concerning the multifaceted neuroprotective characteristics associated with quinoline. In the conducted investigations, the metal chelates and Cu (II) complexes were assessed for their efficacy against toxicities induced by Aβ peptide and H_2_O_2_ in cells. The metal chelates, particularly those involving H_2_dqpyca, demonstrated significant neuroprotective effects against the toxicities induced by Aβ peptide and H_2_O_2_ in SH-SY5Y neuroblastoma cells. A computational study using the crystal structure of rivastigmine-bound acetylcholinesterase (AChE) (PDB ID: 1GQR) suggested a direct interaction between the H_2_dqpyca metal chelate and AChE. Further supporting its potential as a pleiotropic neuroprotective agent against amyloid β-peptide (Aβ) and oxidative stress encountered in cells. In addition, the Cu (II) complex of [Cu^II^(H_2_bqch)Cl_2_].3H_2_O, (H_2_bqch = *N*,*N*′-Bis(8-quinolyl)cyclohexane 1,2-diamine) exhibited neuroprotective properties against H_2_O_2_. In a separate study, a researcher synthesized a series of 4-substituted-2-arylethenylquinoline derivatives and evaluated their anti-Alzheimer activity. Certain compounds bearing an *N*-dimethylaminoalkylamino group at Position 4 of the quinoline scaffold demonstrated potent inhibitory activity against Aβ_1–42_ accumulation and significant antioxidant activity. A Cu (II) Complex 72 derived from one of these ligands exhibited noteworthy properties: it effectively inhibited Aβ aggregation induced by Cu^2+^, separated self-induced Aβ_1–42_ aggregate fibrils, and protected SH-SY5Y cells from Aβ1–42-induced cytotoxicity. Notably, the Cu (II) complex also significantly mitigated scopolamine-induced memory impairment in mice, highlighting its potential as a therapeutic agent for Alzheimer's disease [85]. Another study focused on the design and synthesis of four glycoconjugates based on the quinoline scaffold (Ligands 73a–d), along with their Zn^2+^ and Cu^2+^ complexes. These compounds were investigated as potential antioxidant modifiers and therapeutic agents for neurological disorders, while no of antiproliferative effects were observed on A2780, A549, and SHSY5Y cell lines, the compounds exhibited significant antioxidant activity. Consequently, the focus shifted toward investigating the antioxidant activity of these compounds. In general, the genesis of neurological disorders can be attributed to oxidative stress and the accumulation of proteins. The glycoconjugates demonstrated a substantial capacity to mitigate oxidative stress and address anomalous protein-metal interactions. Notably, the high water solubility of these sugar derivatives allows for investigations to be conducted under physiological conditions [82]. Finally, researchers investigated the antioxidant activity of two innovative biotin 8-hydroxyquinoline and their corresponding metal Complexes 74 involving Mn (II), Co (II), Ni (II), Cu (II), and Zn (II) were conducted in a study by the researchers. The antioxidant effect of these compounds was quantified using 2, 2′-azino-bis-(3-ethylbenzothiazoline-6-sulphonic acid (ABTS) assay, with Trolox as a reference standard. A decrease in endogenous antioxidant levels exacerbates oxidative stress, a key factor in the pathogenesis of Alzheimer's, Parkinson's diseases, and cancer. In vivo studies have demonstrated that these compounds effectively mitigated oxidative damage, suggesting their potential therapeutic value in conditions associated with metal dyshomeostasis and oxidative stress [86] (Figure 16).

### 3.8. Anti-HIV Activity

The production of metal Complex 75 derived from two ligands: (6-benyl-4-oxo-1,4-dihydroquinolin-3-carboxylic acid (HL^1^) and *N*-(4-fluorobenzyl)-5-hydroxy-2-isopropyl-1-methyl-6-oxo-1,6-dihydroxypyrimidine-4-carboxylate (HL^2^)) was investigated by Bacchi et al. as potential anti-HIV drugs. The authors utilized modeling and potentiometric measurements to study the inhibition of the integrase (IN) enzyme. The study also highlighted the significant role of metals in inhibiting integrase (IN). The synthesized metal complexes likely inhibit the enzyme by

• Exchanging metals: This involves the interaction between two metals within the complex, potentially altering the enzyme's activity
• Interacting with the enzyme: The complexes bind to the integrase enzyme, preventing its interaction with viral DNA
• Interacting with the protein: The complexes bind to proteins involved in the integration process, further hindering the enzyme's activity

Results showed that Mg and Mn complexes (L^1^) exhibited four and six times higher activity, respectively, compared to their corresponding ligands. Complexes (L^2^) containing Mg, Mn, Co, and Zn demonstrated comparable efficacy and inhibitory properties to the unbound HL^2^ ligand. Notably, Co (II) complexes with a higher stability constant displayed greater anti-IN activity than other metal complexes in these study [92].

### 3.9. Antioxidant Activity

**Figure 17:**
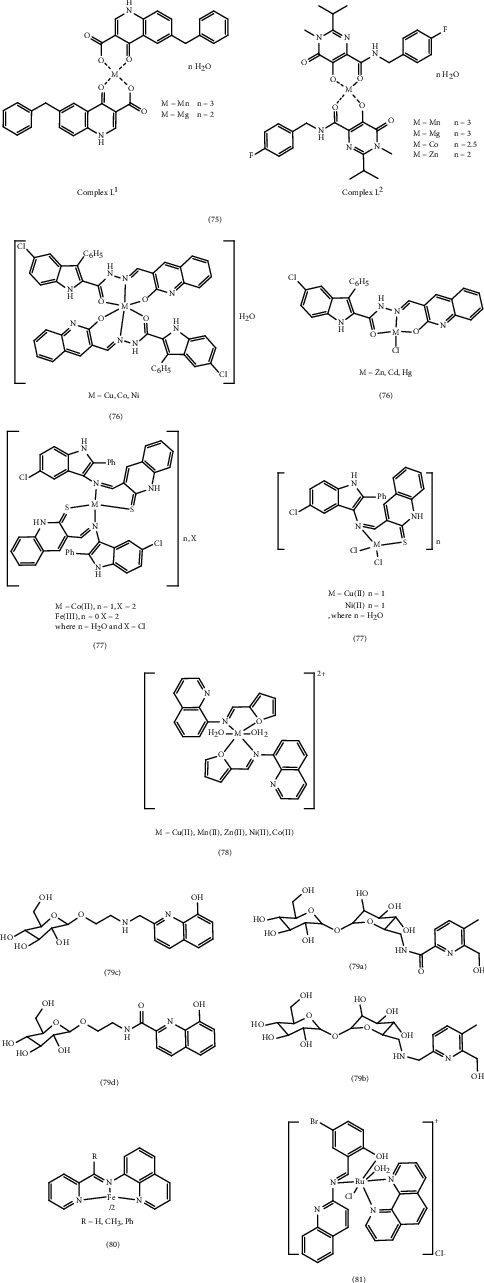
The structure of quinoline metal complexes (75–81) indicating anti-HIV and antioxidant activity.

Mahendra et al. designed and synthesized new complexes of Cu (II), Co (II), Ni (II), Cd (II), and Hg (II) (Complex 76) utilizing quinoline and indole (Schiff base of 5-chloro-3-phenyl-1*H*-indole-2-carboxyhydrazide and 3-formyl-2-hydroxy-1*H*-quinoline (HL)). Their antioxidant activity was assessed by measuring the inhibition of the stable free radical 1, 1-diphenyl-2-picrylhydrazyl (DPPH). Cu (II), Cd (II), Ni (II), and Co (II) complexes exhibited greater inhibitory activity compared to Hg (II) and Zn(II), demonstrating superior antimicrobial and antioxidant activity than the Schiff-based ligand [69]. Vivekanand et al. synthesized Schiff-base quinoline (5-chloro-2- phenyl-1*H*-indol-3-ylimino) methyl) quinoline-2(1*H*)-thione) and their metal complexes (Complex 77). The antioxidant activity of these compounds was assessed using the 1, 1-diphenyl-2-picrylhydrazyl (DPPH) free radical scavenging method. Cu (II), Ni (II), and Zn (II) complexes showed significant and high activity compared to other compounds. Furthermore, DNA cleavage activity was investigated using the calf thymus gel electrophoresis method, revealing that all compounds, except the Schiff-base ligand and Ni (II) complex, were capable of completely cleaving DNA [69]. Shakir et al. examined the synthesis, cytotoxic, antibacterial, and antioxidant properties of the quinoline Schiff-base ligand ((E)-N-(furan-2-yl methylene) quinolin-8-amine) and its complexes with Mn (II), Co (II), Ni (II), Cu (II), and Zn (II) (Complex 78). Using the DPPH method and L-ascorbic acid as a standard, Mn (II) and Zn (II) complexes displayed the highest inhibitory activity. This study highlighted the varied influence of redox properties on the activity of these metal complexes differently [46]. Valentina et al. discovered and synthesized four glycoconjugates based on quinoline (Ligand 79a–d) and their Zn^2+^ and Cu^2+^ complexes as potential antioxidant modifiers for neurological disorders. The antioxidant activity of Zn^2+^ and Cu^2+^ complexes was analyzed using the DPPH radical scavenging method. The presence of Zn^2+^ and Cu^2+^ affects the capacity of compounds, with Zn^2+^ and Cu^2+^ complexes exhibiting significant antioxidant activity. In addition to the presented articles, higher anticancer, antimicrobial, and antioxidant activity of ppaqFe (bis-*N*,*N*′,*N*^″^-[(phenyl-pyridin 2-ylmethylene)-quinolin-8-yl-imine]iron (II)) complex than other complexes and synthetic ligands was observed (Complex 80). Hany Mohamed Abd et al. synthesized a new [4-bromo-2-(quinolin-2-yliminomethyl)-phenol imine-phenanthroline] Ru(III) complex (complexes, 81,) in nano size and used it for potential medicinal applications. They investigated the therapeutic effects of the medicinal combination of this complex, such as the antioxidant and anticancer effect in the treatment of breast cancer and a method for the treatment of microbial infection by one or more types of *E. coli* and *A. flavus* on patients [93] (Figure 17). A summary of the content discussed in this review is also presented in Table 1.

## 4. Conclusion

The versatile nature of quinoline and its analogs as key scaffolds in pharmaceutical synthesis has garnered significant attention within the scientific community. Extensive research into quinoline metal complexes, driven by their enhanced lipophilic properties, has resulted in substantial advancements, enhancing absorption and therapeutic efficacy. These advancements hold great promise in addressing a range of severe diseases, including cancer, AIDS, coronaviruses, neurological disorders, and drug-resistant microbial infections. The innovative synthesis of metal-drug complexes based on quinoline derivatives, combined with modern techniques, demonstrates potent inhibitory potential. Moreover, the utilization of biocompatible drug carriers for targeted delivery to specific cells presents a promising avenue to enhance treatment effectiveness. This review highlights the wide-ranging applications of quinoline-derived compounds and their metal complexes, inspiring innovative approaches in drug synthesis and positioning them to make transformative contributions to modern medicine.

## Figures and Tables

**Table 1:** Information table of the articles mentioned in the review.

**Anticancer activity**					
**Compound number**	**Cell lines**	**Conditions**	**Method/model**	**Duration of treatment**	**Result**

Complex 1	HCT116, A2780	—	MTS	48 h	• A significant increase of cellular ROS (31–97) %
Complex 2	HCT116, A2780	—	MTS	48 h	—
Complex 3	HCT116, A2780	—	MTS	48 h	—
Complex 4	Normal HL-7702, Hep-G2, NCI-H460, and MGC80-3	—	MTT	48 h	• Low cytotoxicity on normal HL-7702 cell lines
Complex 5	Normal HL-7702, Hep-G2, NCI-H460, and MGC80-3	—	MTT	48 h	Low cytotoxicity on normal HL-7702 cell lines
Complex 6	Normal HL-7702, Hep-G2, NCI-H460, and MGC80-3	—	MTT	48 h	• Cytotoxicity assays more than Complexes 4 and 5, highest toxicity • Lower IC_50_ values than cisplatin, and ligands. • Low cytotoxicity on normal HL-7702 cell lines.
Complex 7	Human breast cancer cell lines (MCF-7 and AMJ-13) and normal cells (HBL). Cancer cells (CP3) (WRL-68)	Tem: 37°C	MTT	24 h	• Effective drug against prostate cancer cells and human breast.
Complex 8	Breast cancer cell lines (MDA-MB231)	Tem: 37°C	MTT	24 h	• High cytotoxicity with IC_50_ values of 1.95 and 1.43 μM
Complex 8	Liver cancer cell line (Hep-G2).	Tem: 37°C	MTT	24 h	• Show IC_50_ values of 1.49 and 1.95 μM,
Complex 9	HCT-116, HepG-2, and breast cancer cells (MCF- 7)	—	—	—	• Antiproliferative activities than the free ligand. • Lower cytotoxicity than that of the standard (vinblastine)
Complex 10b	MCF-7	Tem: 37°C	MTT	48 h	• Better anticancer properties on breast cancer cell line (MCF-7) than cisplatin and cyclophosphamide drugs
Complex 11	A549 (non–small-cell lung carcinoma), CH1 (ovarian carcinoma), and SW480 (colon adenocarcinoma),	Tem: 37°C	MTT	24 h	• Higher cytotoxicity and better solubility in compatible environment compared to metal-free ligand
Complex 12	(MCF-7) and lung cancer (A549)	—	MTT	—	• Higher cytotoxicity than that of the other complexes for both cancer cell lines for the dimeric chromium (III) complex
Complex 13	MDA-MB-231, KCL22, HeLa, and PBMC	Tem: 37°C	MTT	48 h	• More promising activity than other synthetic compounds for Mn (II) complex against all cancer cell lines with IC_50_ < 2.10 lM
Complex 14	A427, LCLC-103H, SISO, and 5637	—	—	96 h	• Antiproliferative power of the complexes higher than cisplatin
Complex 15	MCF-7 and HCT-116	Tem: 37°C	—	24 h	• Increase of biological activity with chelation of the Schiff base ligand
Complex 16a and Complex 16b	Adenocarcinoma (A-549/CDDP), (MCF-7)	—	MTT	—	• Significant activity against these two cell lines especially complex, 16b, for cytotoxicity in comparison to cisplatin
Complex 17	Adenocarcinoma cell line (AsPC-1) and acute monocytic leukemia cells (THP-1)	—	—	24 h	• Induce strong apoptosis in THP-1 cells and less activity on AsPC-1 cell line
Complex 18	7404, HeLa, MCF-7, and HepG-2	Tem: 37°C	MTT	72 h	• Potential antiproliferative ability against four human tumor cell lines. • Low IC_50_ values (6.867 and 5.957 μM) compared to cisplatin
Complex 19	Tyrosine kinase (RTK)	—	—	—	• The strong interactions of breast anticancer activity against estrogen receptor
Complex 20	MCF-7	Tem: 37°C	MTT	4 h	• Greater cytotoxicity of Ni (II) complex than the Co (II) complex. • Inhibited the proliferation of cancer cells by the Ni (II) complex with inducing apoptosis via the expression of Caspase-3, the production of reactive oxygen species (ROS), and DNA damage
Complex 21	BEL7404, A549, HeLa, and MCF-7	—	MTT	48 h	• Higher cytotoxicity than oxoglaucine due to the positive synergistic effect with metal-oxoglaucine complexes.
Complex 22	SK-OV-3, BEL-7404, HeLa, Hep-G2, and MGC80-3, HL-7702	—	MTT	—	• Lower inhibitory effects on normal human liver HL-7702 cells compared to cisplatin. Good cytotoxicity against cell lines, such as SK-OV-3 and BEL-7404. • Better performance for the copper complex on MGC80-3 and SK-OV-3 cells than the other complexes and cisplatin, by lower IC_50_ values.

**Antimicrobial activity**					
**Compound number**	**Bacteria and fungi**	**Conditions**	**Method/model**	**Duration of treatment**	**Result**

Complex 23	*B. subtilis*, *S. aureus*, *P. aeruginosa*, and *E. coli*	—	Agar diffusion	—	• Higher efficacy against strains of *B. subtilis* compared to other strains, particularly copper, zinc, and cadmium
Complex 24	*B. subtilis*, *S. aureus*, *C. albicans*, *A. flavus*, *E. coli*, and *P. vulgaris*	Tem: 24°C ± 2°C	Agar diffusion	25–33 h	• The inhibitory power of MSQ metal complexes, free S and Q ligands, and gentamicin standard drugs
Complex 25	*S. aureus*, *E. faecalis*, *P. aeruginosa*, and *E. coli*	—	Agar diffusion	—	• Significant increase in activity against these strains, albeit with a smaller inhibition zone than the standard reference drug
Complex 26a	*S. aureus*, *E. coli*, and *C. albicans*	—	Mueller–Hinton agar and Sabouraud agar	—	• Highest antibacterial and antifungal activity compared to other complexes
Complex 27	*E. coli*, *B. subtilis*, and *A. fumigatus*	—	Disc diffusion and diffusion agar	—	• Significant activities against Gram-positive and Gram-negative bacteria and target fungi. • Highest antimicrobial activity, even more than the approved drug (fluconazole) and ampicillin, for Cu (II) complex compared to the Ni (II) and Co (II) complexes. • More activity complexes than their ligands.
Complex 28	*S. aureus*, *E. coli*, and *A. flavus*	Tem: 37°C Tem: 25°C	Nutrient agar medium and potato dextrose agar (PDA) medium	24 h, 48 h	• Good antifungal and antibacterial activity.
Complex 29	*E. coli*, *C. albicans*, *B. subtilis*, and *A. niger*	—	Agar well diffusion	—	• More inhibitory effect than other complexes and hydrazone ligand alone for copper complex
Complex 30	*S. aureus*, *E. coli*, *P. aeruginosa*, and *C. tropicalis*	—	Disk diffusion	—	• Good inhibition against Gram-positive and negative bacteria for Cu (II) and Co (II) complexes especially against *S. aureus* compared to the ligand. • Also, better activity against *E. coli* bacteria for metal complexes of Zn (II), Cu (II), and Ni (II)
Complex 31	*E. coli* , *E. coli*, and *P. aeruginosa*	Tem: 37°C	Agar plates	48 h	• More active than their ligands, especially copper complexes, significant activity against *A. niger* compared to other bacteria.
Complex 32	*E. coli*, *S. typhi*, *B. subtilis*, *S. aureus*, *C. albicans*, , *A. niger*	Tem: 37°C	Muller–Hinton agar media	24 h	• Increasing the lipophilicity of the metal chelate and cutting the genomic DNA of *E. coli* by Cu (II) complex
Complex 33	*E. aerogenes*, *P. aeruginosa*, *A. niger*, and *A. flavus*	Tem: 37°C	Agar well diffusion	18 h	• Superior antimicrobial activity of the metal complexes compared to the free ligand
Complex 34	*C. albicans*, *B. thibromine*, N. Sp, *A. fumigatus*, and *R. nigricans*	—	Potato dextrose agar (PDA) medium	120 h	• Highest antifungal activity of the Schiff base ligand. • Increasing antifungal activity by substituting the phenyl rings with chlorine.
Complex 35	*E. coli*, *S. aureus*, *A. niger*, and *P. chrysogenum*	Tem: 37°C	Potato dextrose agar diffusion	24 and 48 h	• More bactericidal activity of the copper metal complexes than the other complexes and ligands
Complex 36	*S. aureus*, *P. aeruginosa*, *A. niger*, and *A. flavus*	Tem: 37°C	Cellulose dextrose agar (SDA)	24 h for bactria 48 h for fungi	• More effective against *S. aureus* and *P. aeruginosa* for Cu (II), Co (II), and Fe (III) complexes than the other complexes. • More effective against *A. niger* and *A. flavus* for Cu (II) and Co (II) complexes than their synthetic counterparts.
Complex 37	*S. aureus*, *E. coli*, *E. cloacae*, and *B. subtilis*	Tem: 37°C	Nutrient agar diffusion	24 h for bactria 48 h for fungi	• Have antibacterial activity for Schiff base ligand and its zinc and copper complex
Complex 38	*S. typhimurium*, *P. aeruginosa*, Vibriocholera, *S. aureus*, *L. monocytogenes*, *E. coli*, and *C. albicans*	Tem: 37°C	—	12–18 h	• Significant effect of chelation on antimicrobial activity of complexes
Complex 40	*S. aureus* and *E. coli*	—	Sabouraud dextrose agar and Müller–Hinton agar	—	• Stronger antibacterial activity of the Cu (II) complex compared to the Zn (II) complex and its ligand and the standard antibiotic penicillin.
Complex 41	Bacillus sp, Klebsiella sp, Proteus sp, Klebsiella sp, and Proteus sp	Tem: 25°C	Agar disk diffusion	24 h	• Better results than the introduced antibiotic drug (ciproH).
Complex 42	*E. faecalis* *C. albicans* *B. subtilis* *K. pneumonia*	—	—	—	• Increase antimicrobial activity due to the lipophilicity, stability, steric, and electronic properties of the zinc (II) complexes.
Complex 43	*S. pneumonia*, *B. subtilis*, *P. aeruginosa*, and *E. coli*	Tem: 37°C for bactria, Tem: 25°C for fungi	Sabouraud dextrose agar medium	24 h for bactria 48 h for fungi	• Lower activity of metal complexes against *S. pneumoniae* in comparison to the Schiff base ligand. • More effective than the Schiff base ligand against *B. subtilis*. • Less effective for Cr (III) and Fe (III) complexes than the Schiff base ligand against the Gram-negative bacterium *P. aeruginosa*. Compared to the Schiff base ligand, • Antifungal activity, all of the compounds except for the Cr (III) complex and the Ni (II) complex outperformed the Schiff base ligand.
Complex 44	*P. aeruginosa*, *E. coli*, *B. subtilis*, and *S. aureus*	—	—	—	• Better antibacterial activity for complexes than free ligands or metal salts alone compared to reference antibiotics streptomycin, penicillin, and ciprofloxacin. • High antibacterial activity against *S. aureus* for Zn (II) and Cd (II) complexes with IC_50_ = 0.57 μg/mL and 0.51 μg/mL compared to positive controls.
Complex 45	*E. coli*	—	—	—	• Significant antibacterial activity against all bacteria of the complexes. • Not inhibitory activity the [(Ni)_2_(Q)_2_(KL)(H_2_O)_4_] complex against *E. coli*.
Complexes 46, 47, and 48	*S. pneumonia*, *B. subtilis*, *P. aeruginosa*, *E. coli*, and *C. albicans*	—	Agar diffusion	—	• Greatest potency antibacterial activity of ligand L^c^ against *S. pneumonia*, *B. subtilis*, *P. aeruginosa*, and *E. coli*. • Antifungal activity of the Ni (II) and Co (II) complexes of ligand L^c^ (ligand, 46c) (complex, 47) against *A. fumigates* and have antifungal activity of Ni (II), Co(II), and Fe(II) complexes of ligand L^b^ (ligand, 46b) (complexes, 48) against *C. albicans*
Complex 49	*B. cereus*, *S. aureus*, *S. lutea*, *E. coli*, *S. shiga*, and *P. aeruginosa*	—	—	—	• Greater inhibitory potency and sensitivity of complexes in comparison to the standard kanamycin. • Superior efficacy of complexes compared to the individual ligands and standard drug
Complex 50	*S. aureus*, *B. subtillis*, *E. coli*, P*. aeruginosa*, *A. niger*, and *A. flavus*	—	—	—	• Superior and statistically significant antibacterial efficacy of ligand compared to both the metal complexes and ciprofloxacin. • Superior activity of Fe (II) and Cu (II) complexes compared to the other complexes.
Complex 51	*A. niger*, *C. albicans*, *E. coli*, *S. typhi*, *S. aureus*, and *B. subtilis*	Tem: 37°C for bactria, Tem: 30°C for fungi	Nutrient agar, potato dextrose agar	24 h for bactria 72 h for fungi	• Higher antimicrobial activity of metal complexes, especially Cu (II) complexes than the free ligand against Gram-positive and Gram-negative bacteria and two yeasts (*A. niger* and *C. albicans*). • Significant effect of the octahedral structure of the complexes on antimicrobial activity.
Complex 52	*P. aeruginosa*, *B. subtilis*, *E. coli*, and *S. aureus*	—	Müller–Hinton agar	—	• Better activity of Cd and Co (II) complexes on Gram-positive bacteria than Gram-negative bacteria. • Only effect of Ag (I) complex on Gram-negative bacteria *P. aeruginosa* with IC_50_ = 6.74 μg/mL.
Complex 53	*P. aeruginosa*, *S. aureus*, *A. niger*, and *C. albicans*	Tem: 35°C for bactria, Tem: 28°C for fungi	Nutrient agar and potato dextrose	24 h for bacteria 48 h for fungi	• Greater inhibitory activity of metal complexes than the synthetic ligand compared to chloramphenicol and fluconazole standards
Complex 54	*S. marcescens* *E. coli* *M. luteus* *A. flavus* *G. candidum* *F. oxysporum*	—	—	—	• Strongest inhibition for BSQPd complex among all compounds
Complex 55	*P. aeruginosa*, *E. coli*, *E. faecalis*, *S. aureus*, and *C. albicans*	Tem: 37°C	Agar Müeller–Hinton medium	48 h	• Low cytotoxicity toward blood cells and moderate antimicrobial effects of compounds. • Efficacy against both Gram-positive and Gram-negative bacteria notably Complex 55

**Anthelmintic activity**					
**Compound number**	**Earthworms**	**Conditions**	**Method/model**	**Duration of treatment**	**Result**

Complex 51	Pheretima posthuma	Warm water (50°C)	—	—	• Significant activity with complete paralysis and death of worms with Cu (II) and Zn (II) complexes compare of the albendazole standard

**DNA cleavage studies**					
**Compound number**	**Cells**	**Conditions**	**Method/model**	**Duration of treatment**	**Result**

Complex 56	*E. coli*	Constant electricity: 50V	Electrophoresis method agarose gel	30 min	• Growth inhibition of the pathogenic organism of Co (II) and Ni (II) complexes with genome cleavage and significant role in the cleavage reaction of isolated DNA
Complex 57	Plasmid DNA (pUC 19) and calf thymus DNA	pH: 7.0 Tem: r.t Constant electricity: 50V	Gel electrophoresis and UV spectroscopy	30–45 min	• Maximum cleavage power and intensity of DNA lines of Ni (II), Cu (II), and Zn (II) complexes. • Higher binding power of complexes and DNA than the binding power of ligands, especially Cu-AHQMBSH complex.
Complex 58	Calf thymus DNA (CT-DNA)	UV absorbance at 260 and 280 nm. Range of scan 220–320 nm with a speed of 10 nm min^−1^ at 25°C. Fluorescence spectra wavelength at 530 nm and an emission wavelength of 600 nm at room temperature.	Electronic absorption spectra circular dichroism (CD) spectra and fluorescence spectra	—	• Stronger connection of Co (II) complex with DNA through DNA grooves placement and DNA cleavage. • More important and stronger binding to DNA of complex 58b, compared to complex 58a.
Complex 59	DNA	UV-vis and photoluminescence	Solvothermal	—	• The changes of the EtBr-DNA system and the quenching rate constant K by adding the complex to DNA, with strong bond between the complex and DNA.
Complex 60	CT-DNA	Measuring of purity at absorbanceat 260 nm and at 280 nm. Use of the buffer [5 mM tris (hydroxymethyl) aminomethane, pH 7.2, 50 mM NaCl] for the absorption and viscosity.	Ultraviolet-visible absorption spectra	—	• Higher affinity of the bathochromic and hypochromic alterations in the absorption spectrum of the Co (II) complex toward CT-DNA compared to the Ni (II) complex. • Increase in the viscosity of DNA fragments the presence of these complexes.
Complex 61	Plasmid pBR322 DNA	—	Agarose gel electrophoresis technique	—	• Observe indicative of the influence of metal complexes and ligands on DNA.
Complexes, 62, 63, and 64	B-DNA	—	Agarose gel electrophoresis	90 min	• Strategically positioned within the active site of B-DNA for complexes, 62–64, specifically within the minor groove region. • Binding affinity with HSA surpassed that of other copper (II) complexes.
Complex 65	CT-DNA	—	Agarose gel electrophoresis assay, UV-vis spectroscopy, fluorescence analysis, circular dichroism (CD) spectroscopy, and viscosity measurements.	—	• Inhibition of topoisomerase I (TOPO I) by metal complexes
Complex 66	CT-DNA	In 0.01 M tris buffer (pH = 7.5, tem: 25°C)	Spectrophotometric method and Hydrodynamic measurements	—	• Clear indicators of the interactions between the complexes and DNA

**Photodynamic activity**					
**Compound number**	**Cells**	**Conditions**	**Method/model**	**Duration of treatment**	**Result**

Complex 67	HepG2 human hepatocarcinoma	Measuring their fluorescence at *λ* = 670 nm, 80 mW cm^−2^, and 1.5 J cm^−2^.	Fluorescence Microscopy. Confocal laser scanning microscopy	—	• High phototoxicity of HepG2 cells with IC_50_ values of 0.02–0.05 μM in the presence of light, low accumulation, and high cellular uptake with measuring phthalocyanines act on mitochondria and lysosomes of cells. • Very useful antitumor drugs for photodynamic therapy of monosubstituted phthalocyanines

**Antitubercular activity**					
**Compound number**	**Cells**	**Conditions**	**Method/model**	**Duration of treatment**	**Result**

Complex 68	Mycobacterium tuberculosis strain (H37 RV)	—	SAR study	—	• Stop cell growth by stopping the DNA gyrase enzyme from working by the metal complexes. • Substantial impact of heterocyclic ring on the growth inhibition of Mycobacterium tuberculosis
Complex 69	Mycobacterium tuberculosis	Tem: 37°C	Alamar blue (MABA) microplate. MIC	24 h	• Superior antibacterial efficacy against Mycobacterium tuberculosis, namely, the H37 RV strain for copper (II) complexes and zinc (II) complexes.
Complex 70	M. tuberculosis (strain H37 RV)	—	Blue almar technique, fluorescence, and ultraviolet (UV)	—	• Anti-tuberculosis activity, particularly the complexes (*X* = Cl, br, Ar = pyridine).

**Neuroprotective studies**					
**Compound number**	**Cells**	**Conditions**	**Method/model**	**Duration of treatment**	**Result**

Complex 71	SH-SY5Y neuroblastoma	—	MTS assay	4.5 h	• Neuroprotective activity against amyloid β-peptide (Aβ) and oxidative stress in cells. • Neuroprotective properties of the Cu (II) complex of [CuII(H2bqch)Cl2].3H2O, (H2bqch = *N*,*N*′ -Bis(8-quinolyl) cyclohexane 1,2-diamine) against H_2_O_2_
Complex 72	SH-SY5Y	Tem: 37°C	MTT	24 h and 48 h	• Further investigation of Cu (II) complex as a drug for treating Alzheimer's disease.
Complex 73	A2780, A549, and SHSY5Y	—	—	—	• Aiming of the Zn^2+^ and Cu^2+^ complexes to act as antioxidant modifiers and suitable therapeutic agents for treating neurological disorders. • Antiproliferative activity of none of the Cu^2+^ complex.

**Anti HIV activity**					
**Compound number**	**Cells**	**Conditions**	**Method/model**	**Duration of treatment**	**Result**

Complex 75	Integrase (IN) enzyme	pH = 7.2 Tem: 30°C	—	30 min	• The activity of Mg and Mn complexes (L^1^) compared to their respective ligands. • The efficacy and inhibitory properties of the complexes (L^2^) containing mg, Mn, Co, and Zn comparable to HL^2^ ligand. • Higher stability constant demonstrates greater anti-IN activity of the Co (II) complexes than metal complexes.

**Antioxidant activity**					
**Compound number**	**Cells**	**Conditions**	**Method/model**	**Duration of treatment**	**Result**

Complex 76	Radical 1, 1-diphenyl-2-picrylhydrazyl (DPPH)	Absorbance of test solutions: at 517 nm.	DPPH	—	• Greater inhibitory of Cu (II), Cd (II), Ni (II), and Co (II) complexes activity than Hg (II) and Zn(II). • Greater antimicrobial and antioxidant activity of the complexes than the Schiff-based ligand.
Complex 77	DPPH	Absorbance of test solutions: at 517 nm	DPPH	30 min	• Significant and high activity of the Cu (II), Ni (II), and Zn (II) complexes, in comparison to the other compounds.
Complex 78	DPPH	—	DPPH	—	• Highest inhibitory activity of the Mn (II) and Zn (II) complexes. • Affect the activity of metal complexes.
Complex 79	DPPH	Absorbance of test solutions: at 515 nm	DPPH	1 h	• Significant antioxidant activity of the Zn^2+^ and Cu^2+^ complexes

Abbreviation: Tem, temperature.

## Data Availability

The data used to support the findings of the study can be obtained from the journal site.

## Nomenclature

HIV: Human immunodeficiency virus
DNA: Deoxyribonucleic acid
MIC: Minimum inhibitory concentration
IC_50_: Half maximal inhibitory concentration
MTT: Microturbine technology
AIDS: Acquired immunodeficiency syndrome
COVID-19: Coronavirus disease 2019
CFSE: Crystal field stabilization energy
dtpy: 2,6-di(thiazol-2-yl) pyridine
dppy: 2,6-di(pyrazin-2-yl) pyridine
terpy: 2,2′:6′,2″- terpyridine
ROS: Reactive oxygen species
HCT116: Colorectal cancer
HeLa: Cervical carcinoma
HL-7702: Normal human liver cell
Hep-G2: Human liver cancer cell
NCI-H460: Human non–small-cell lung adenocarcinoma
MGC80-3: Human gastric adenocarcinoma cell
8-HQ: 8-Hydroxyquinoline
MCF-7: Human breast adenocarcinoma
AMJ-13: Human breast cancer cell line
HBL: Normal cells
CP_3_: Cancer cells
WRL-68: Human hepatic cell line
MSQ: Metal complexes including salin (S) and 8-hydroxyquinoline (Q)
MDA-MB231: Human metastatic breast cancer cell line
MOE: Molecular operating environment
PDB: Protein data bank
A549: Non–small-cell lung carcinoma
CH1: Ovarian carcinoma
SW480: Colon adenocarcinoma
H‐DPPMHQ: Methylene dehydrazineyl quinoline
KCL22: Blood lymphoid carcinoma
PBMC: Peripheral blood mononuclear cells
A427: Lung cancer cell line
LCLC-103H: Large cell lung cancer
SISO: Uterine adenocarcinoma
5637: Human bladder
A-549/CDDP: Adenocarcinoma
CT-DNA: Circulating tumor DNA
Hqasesc: 2-Quinoline carboxaldehyde seleno semicarbazone ligands
HSA: Human serum albumin
AsPC-1: Pancreatic adenocarcinoma cell
THP-1: Human leukemia monocytic cell
OG: Oxoglaucine
BEL7404: Human hepatoma cells
HL-7702: Normal liver cells
SK-OV-3: Ovarian cancer cell
MDA MB-231: Human breast adenocarcinoma
ATCC HTB-26: Breast tumor cells
LMCT: Ligand-to-metal charge transfers
DFT: Density functional theory
ICT: Intramolecular charge transfer
SDA: Cellulose dextrose agar method
MTCC-1881: Yeasts (*Aspergillus niger*)
MTCC-227: Yeasts (*Candida albicans*)
CLSI: Clinical Laboratory and Standards Institute
TOPO I: Topoisomerase I
MABA: Alamar blue microplate method
H37 RV: Mycobacterium tuberculosis strain
UV: Ultraviolet
Hbpq: *N*-(8-quinolyl) pyridine-2-carboxamide
H_2_dqpyca: *N* ^2^,*N*^6^-Di(quinolin-8-yl) pyridine-2,6-dicarboxamide
H_2_bqch: *N,N*′*-*Bis(8-quinolyl) cyclohexane-1,2-diamine
SH-SY5Y: Neuroblastoma cells
AChE: Acetylcholinesterase
ABTS: 2, 2′-Azino-bis-(3-ethylbenzothiazoline-6-sulphonic acid
DPPH: 1, 1-diphenyl-2-picrylhydrazyl

## References

[1] Kalaria P. N., Karad S. C., Raval D. K. (2018). A Review on Diverse Heterocyclic Compounds as the Privileged Scaffolds in Antimalarial Drug Discovery. *European Journal of Medicinal Chemistry*.

[2] Hu Y. Q., Gao C., Zhang S. (2017). Quinoline Hybrids and Their Antiplasmodial and Antimalarial Activities. *European Journal of Medicinal Chemistry*.

[3] Mantu D., Antoci V., Moldoveanu C., Zbancioc G., Mangalagiu I. I. (2016). Hybrid Imidazole (Benzimidazole)/Pyridine (Quinoline) Derivatives and Evaluation of Their Anticancer and Antimycobacterial Activity. *Journal of Enzyme Inhibition and Medicinal Chemistry*.

[4] Zhong F., Geng G., Chen B. (2015). Identification of Benzenesulfonamide Quinoline Derivatives as Potent HIV-1 Replication Inhibitors Targeting Rev Protein. *Organic and Biomolecular Chemistry*.

[5] Sen C., Sahoo T., Singh H., Suresh E., Ghosh S. C. (2019). Visible Light-Promoted Photocatalytic C-5 Carboxylation of 8-Aminoquinoline Amides and Sulfonamides via a Single Electron Transfer Pathway. *Journal of Organic Chemistry*.

[6] Guarner J. (2020). *Three Emerging Coronaviruses in Two Decades: The Story of SARS, MERS, and now COVID-19*.

[7] Zhao R., Lu Z., Yang J., Zhang L., Li Y., Zhang X. (2020). Drug Delivery System in the Treatment of Diabetes Mellitus. *Frontiers in Bioengineering and Biotechnology*.

[8] Zhang S. S., Tan Q. W., Guan L. P. (2021). Antioxidant, Anti-Inflammatory, Antibacterial, and Analgesic Activities and Mechanisms of Quinolines, Indoles and Related Derivatives. *Mini Reviews in Medicinal Chemistry*.

[9] Malghani Z., Khan A.-U., Faheem M. (2020). Molecular Docking, Antioxidant, Anticancer and Antileishmanial Effects of Newly Synthesized Quinoline Derivatives. *Anti-Cancer Agents in Medicinal Chemistry*.

[10] Owais M., Kumar A., Hasan S. M. (2024). Quinoline Derivatives as Promising Scaffolds for Antitubercular Activity: A Comprehensive Review. *Mini Reviews in Medicinal Chemistry*.

[11] Keri R. S., Patil S. A. (2014). Quinoline: a Promising Antitubercular Target. *Biomedicine & Pharmacotherapy*.

[12] Chen C. S., Lai S. Y., Hsu P. S. (2002). Design, Synthesis and Biological Evaluation of Heterocycle-Conjugated Styrene Derivatives as Protein Tyrosine Kinase Inhibitors and Free Radical Scavengers. *Chinese Pharmaceutical Journal*.

[13] Zheng H., Weiner L. M., Bar-Am O. (2005). Design, Synthesis, and Evaluation of Novel Bifunctional Iron-Chelators as Potential Agents for Neuroprotection in Alzheimer’s, Parkinson’s, and Other Neurodegenerative Diseases. *Bioorganic & Medicinal Chemistry*.

[14] Zhang H., Han L. F., Zachariasse K. A., Jiang Y. B. (2005). 8-Hydroxyquinoline Benzoates as Highly Sensitive Fluorescent Chemosensors for Transition Metal Ions. *Organic Letters*.

[15] Shakour N., Hoseinpoor S., Rajabian F. (2024). Discovery of Non-Peptide GLP-1r Natural Agonists for Enhancing Coronary Safety in Type 2 Diabetes Patients. *Journal of Biomolecular Structure and Dynamics*.

[16] Hoseinpoor S., Chamani J., Saberi M., Iranshahi M., Amiri-Tehranizadeh Z., Shakour N. (2024). PPARG Modulation by Bioactive Compounds from Salvia Officinalis and Glycyrrhiza Glabra in Type 2 Diabetes Management: A In Silico Study. *Innovation and Emerging Technologies*.

[17] Song K. C., Kim J. S., Park S. M., Chung K.-C., Ahn S., Chang S. K. (2006). Fluorogenic Hg2+-Selective Chemodosimeter Derived From 8-Hydroxyquinoline. *Organic Letters*.

[18] Kandeel M., Al-Nazawi M. (2020). Virtual Screening and Repurposing of FDA Approved Drugs Against COVID-19 Main Protease. *Life Sciences*.

[19] Vellingiri B., Jayaramayya K., Iyer M. (2020). COVID-19: A Promising Cure for the Global Panic. *Science of the Total Environment*.

[20] Heiskanen J. P., Omar W. A. E., Ylikunnari M. K., Haavisto K. M., Juan M. J., Hormi O. E. O. (2007). Synthesis of 4-Alkoxy-8-Hydroxyquinolines. *Journal of Organic Chemistry*.

[21] Kim I., Kim C. H., Kim J. H. (2004). Pyrrolidine Dithiocarbamate and Zinc Inhibit Proteasome-Dependent Proteolysis. *Experimental Cell Research*.

[22] Xiao Y. A. N., Chen D. I., Zhang X. I. A. (2010). Molecular Study on Copper-Mediated Tumor Proteasome Inhibition and Cell Death. *International Journal of Oncology*.

[23] Azimi S., Merza M. S., Ghasemi F. (2023). Green and Rapid and Instrumental One-Pot Method for the Synthesis of Imidazolines Having Potential Anti-SARS-CoV-2 Main Protease Activity. *Sustainable Chemistry and Pharmacy*.

[24] Chiara F., Gambalunga A., Sciacovelli M. (2012). Chemotherapeutic Induction of Mitochondrial Oxidative Stress Activates GSK-3α/β and Bax, Leading to Permeability Transition Pore Opening and Tumor Cell Death. *Cell Death & Disease*.

[25] Verani C. N. (2012). Metal Complexes as Inhibitors of the 26S Proteasome in Tumor Cells. *Journal of Inorganic Biochemistry*.

[26] Ikotun O. F., Higbee E. M., Ouellette W., Doyle R. P. (2009). Pyrophosphate-Bridged Complexes With Picomolar Toxicity. *Journal of Inorganic Biochemistry*.

[27] Chen H. L., Chang C. Y., Lee H. T. (2009). Synthesis and Pharmacological Exploitation of Clioquinol-Derived Copper-Binding Apoptosis Inducers Triggering Reactive Oxygen Species Generation and MAPK Pathway Activation. *Bioorganic & Medicinal Chemistry*.

[28] Jungwirth U., Kowol C. R., Keppler B. K., Hartinger C. G., Berger W., Heffeter P. (2011). Anticancer Activity of Metal Complexes: Involvement of Redox Processes.

[29] Anbu S., Kandaswamy M. (2012). DNA Binding, DNA Hydrolase and Phosphatase like Activity of New Polyaza Macrobicyclic Binuclear Copper(II), Nickel(II) and Zinc(II) Complexes. *Inorganica Chimica Acta*.

[30] McMillin D. R., Kirchhoff J. R., Goodwin K. V. (1985). Exciplex Quenching of Photo-Excitd Copper Complexes. *Coordination Chemistry Reviews*.

[31] Chifotides H. T., Dunbar K. R. (2005). Interactions of Metal-Metal-Bonded Antitumor Active Complexes With DNA Fragments and DNA. *Accounts of Chemical Research*.

[32] Fernández M. J., Wilson B., Palacios M., Rodrigo M.-M., Grant K. B., Lorente A. (2007). Copper-Activated DNA Photocleavage by a Pyridine-Linked Bis-Acridine Intercalator. *Bioconjugate Chemistry*.

[33] Lighvan Z. M., Abedi A., Bordar M. (2012). Novel Mononuclear Zinc Complexes With 2,2′-Dimethyl-4,4′-Bithiazole: Synthesis, Crystal Structure and DNA-Binding Studies. *Polyhedron*.

[34] Kostova I., Balkansky S. (2013). Metal Complexes of Biologically Active Ligands as Potential Antioxidants. *Current Medicinal Chemistry*.

[35] Mandewale M. C., Thorat B., Shelke D., Yamgar R. (2015). Synthesis and Biological Evaluation of New Hydrazone Derivatives of Quinoline and Their Cu (II) and Zn (II) Complexes Against *Mycobacterium tuberculosis*. *Bioinorganic Chemistry and Applications*.

[36] Choroba K., Machura B., Kula S. (2019). Copper (II) Complexes with 2, 2′: 6′, 2′′-terpyridine, 2, 6-di (Thiazol-2-yl) Pyridine and 2, 6-di (Pyrazin-2-yl) Pyridine Substituted With Quinolines. Synthesis, Structure, Antiproliferative Activity, and Catalytic Activity in the Oxidation of Alkanes and Alcohols With Peroxides. *Dalton Transactions*.

[37] Hu K., Liu C., Li J., Liang F. (2018). Copper (II) Complexes Based on Quinoline-Derived Schiff-Base Ligands: Synthesis, Characterization, HSA/DNA Binding Ability, and Anticancer Activity. *MedChemComm*.

[38] Reddy T. R., Ramkumar J., Chandramouleeswaran S., Reddy A. V. R. (2010). Selective Transport of Copper Across a Bulk Liquid Membrane Using 8-hydroxy Quinoline as Carrier. *Journal of Membrane Science*.

[39] Al-Farhan B. S., Basha M. T., Abdel Rahman L. H. (2021). Synthesis, DFT Calculations, Antiproliferative, Bactericidal Activity and Molecular Docking of Novel Mixed-Ligand Salen/8-Hydroxyquinoline Metal Complexes. *Molecules*.

[40] Al-Abdulkarim H. A., El-khatib R. M., Aljohani F. S. (2021). Optimization for Synthesized Quinoline-Based Cr^3+^, VO^2+^, Zn^2+^ and Pd^2+^ Complexes: DNA Interaction, Biological Assay and In-Silico Treatments for Verification. *Journal of Molecular Liquids*.

[41] Dalzon B., Bons J., Diemer H. (2019). A Proteomic View of Cellular Responses to Anticancer Quinoline-Copper Complexes. *Proteomes*.

[42] dos Santos Chagas C., Fonseca F. L. A., Bagatin I. A. (2019). Quinoline-Derivative Coordination Compounds as Potential Applications to Antibacterial and Antineoplasic Drugs. *Materials Science and Engineering: C*.

[43] Primik M. F., Göschl S., Meier S. M. (2013). Dicopper (II) and Dizinc (II) Complexes With Nonsymmetric Dinucleating Ligands Based on Indolo [3, 2-c] Quinolines: Synthesis, Structure, Cytotoxicity, and Intracellular Distribution. *Inorganic Chemistry*.

[44] Ammar R. A., Alaghaz A. N. M. A., Alturiqi A. S. (2018). New Dimeric Schiff Base Quinoline Complexes: Synthesis, Spectral Characterization, Electrochemistry and Cytotoxicity. *Applied Organometallic Chemistry*.

[45] Azimi S. G., Bagherzade G., Saberi M. R., Amiri Tehranizadeh Z. (2023). Discovery of New Ligand With Quinoline Scaffold as Potent Allosteric Inhibitor of HIV‐1 and its Copper Complexes as a Powerful Catalyst for the Synthesis of Chiral Benzimidazole Derivatives, and In Silico Anti‐HIV‐1 Studies. *Bioinorganic Chemistry and Applications*.

[46] Shakir M., Hanif S., Sherwani M. A., Mohammad O., Al-Resayes S. I. (2015). Pharmacologically Significant Complexes of Mn (II), Co (II), Ni (II), Cu (II) and Zn (II) of Novel Schiff Base ligand,(E)-*N*-(furan-2-yl Methylene) Quinolin-8-Amine: Synthesis, Spectral, XRD, SEM, Antimicrobial, Antioxidant and In Vitro Cytotoxic Studies. *Journal of Molecular Structure*.

[47] Martín-Santos C., Michelucci E., Marzo T. (2015). José Alemán. Gold(III) Complexes with Hydroxyquinoline, Aminoquinoline and Quinoline Ligands: Synthesis, Cytotoxicity, DNA and Protein Binding Studies. *Journal of Inorganic Biochemistry*.

[48] Abd El‐Halim H. F., Mohamed G. G., Anwar M. N. (2018). Antimicrobial and Anticancer Activities of Schiff Base Ligand and its Transition Metal Mixed Ligand Complexes With Heterocyclic Base. *Applied Organometallic Chemistry*.

[49] Fan X., Dong J., Min R. (2013). Cobalt (II) Complexes With Thiosemicarbazone as Potential Antitumor Agents: Synthesis, Crystal Structures, DNA Interactions, and Cytotoxicity. *Journal of Coordination Chemistry*.

[50] Filipović N. R., Bjelogrlić S., Marinković A. (2015). Zn (II) Complex With 2-quinolinecarboxaldehyde Selenosemicarbazone: Synthesis, Structure, Interaction Studies With DNA/HSA, Molecular Docking and Caspase-8 And-9 Independent Apoptose Induction. *RSC Advances*.

[51] Lu J., Li J. L., Sun Q. (2014). Synthesis, Characterization, and Biological Activities of Two Cu (II) and Zn (II) Complexes With One Polyquinoline Ligand. *Spectrochimica Acta Part A: Molecular and Biomolecular Spectroscopy*.

[52] Lokesh M. R., Krishnamurthy G., Bhojyanaik H. S., Shashikumar N. D., Krishna P. M. (2014). DNA Binding, In Silico Docking and In Vitro Biological Screening of Some Transition Metal Complexes of Schiff Base Ligand as Potential Blockers of Cancer Causing Receptors. *International Journal of TechnoChem Research*.

[53] Lamani D. S., Badiger S. G., Venugopala Reddy K. R., Bhojya Naik H. S. (2018). Macrocyclic Complexes: Synthesis, Characterization, Antitumor and DNA Binding Studies. *Nucleosides, Nucleotides & Nucleic Acids*.

[54] Chen Z. F., Shi Y. F., Liu Y. C. (2012). TCM Active Ingredient Oxoglaucine Metal Complexes: Crystal Structure, Cytotoxicity, and Interaction With DNA. *Inorganic Chemistry*.

[55] Zou B. Q., Lu X., Qin Q. P. (2017). Three Novel Transition Metal Complexes of 6-Methyl-2-Oxo-Quinoline-3-Carbaldehyde Thiosemicarbazone: Synthesis, Crystal Structure, Cytotoxicity, and Mechanism of Action. *RSC Advances*.

[56] Damit N. S. H. H., Hamid M. H. S. A., Rahman N. S. R. H. A., Ilias S. N. H. H., Keasberry N. A. (2021). Synthesis, Structural Characterisation and Antibacterial Activities of Lead(II) and Some Transition Metal Complexes Derived From Quinoline-2-Carboxaldehyde 4-Methyl-3-Thiosemicarbazone. *Inorganica Chimica Acta*.

[57] Ashram M., Al-Mazaideh G. M., Al-Zereini W., Al-Mustafa A., Mizyed S. (2019). Synthesis, Complexation and Biological Effects Studies of New Thiacrown Ethers Derived Quinoline: Part I. *Journal of Sulfur Chemistry*.

[58] Althobiti H. A., Zabin S. A. (2020). New Schiff Bases of 2-(quinolin-8-Yloxy)acetohydrazide and Their Cu(II), and Zn(ii) Metal Complexes: Their In Vitro Antimicrobial Potentials and In Silico Physicochemical and Pharmacokinetics Properties. *Journal Open Chemistry*.

[59] Diaconu D., Mangalagiu V., Amariucai-Mantu D., Antoci V., Giuroiu C. L., Mangalagiu I. I. (2020). Hybrid Quinoline-Sulfonamide Complexes (M(2+)) Derivatives With Antimicrobial Activity. *Molecules*.

[60] Ali I. A. I., El-Sakka S. S. A., Soliman M. H. A., Mohamed O. E. A. (2019). In Silico, In Vitro and Docking Applications for Some Novel Complexes Derived From New Quinoline Derivatives. *Journal of Molecular Structure*.

[61] Bhuvanesh N., Suresh S., Velmurugan K., Thamilselvan A., Nandhakumar R. (2020). Quinoline Based Probes: Large Blue Shifted Fluorescent and Electrochemical Sensing of Cerium Ion and its Biological Applications. *Journal of Photochemistry and Photobiology A: Chemistry*.

[62] El-Saied F., Shakdofa M., Al-Hakimi A., Shakdofa A. (2020). Transition Metal Complexes Derived From N′‐(4‐fluorobenzylidene)‐2‐(quinolin‐2‐yloxy) Acetohydrazide: Synthesis, Structural Characterization, and Biocidal Evaluation. *Applied Organometallic Chemistry*.

[63] Abdul S. S. A. A. J., Nasser S. R., Brindha G. (2014). Synthesis, Spectral Studies and Antibacterial Activities of 8-hydroxyquinoline Derivative and its Metal Complexes. *Chemical Science Transactions*.

[64] Kulkarni N. V., Hegde G. S., Kurdekar G. S., Budagumpi S., Sathisha M. P., Revankar V. K. (2010). Spectroscopy, Electrochemistry, and Structure of 3d-Transition Metal Complexes of Thiosemicarbazones With Quinoline Core: Evaluation of Antimicrobial Property. *Spectroscopy Letters*.

[65] Karekal M. R., Biradar V., Hire Mathada B., Synthesis M. (2013). Characterization, Antimicrobial, DNA Cleavage, and Antioxidant Studies of Some Metal Complexes Derived From Schiff Base Containing Indole and Quinoline Moieties. *Bioinorganic Chemistry and Applications*.

[66] Yernale N. G., Hire Mathada B., Synthesis M. (2014). Characterization, Antimicrobial, DNA Cleavage, and In Vitro Cytotoxic Studies of Some Metal Complexes of Schiff Base Ligand Derived From Thiazole and Quinoline Moiety. *Bioinorganic Chemistry and Applications*.

[67] Vashi R., Shelat C., Patel H. (2010). Synthesis and Antifungal Activity of 6-bromo-2[(4-(2,3- Dichloropheyl)) Piperazine-1-Yl)methyl]-3-[8-Hydroxy Quinoline-5-Yl]-3-Quinazolin-4-One Ligand and Itstransition Metal Chelates. *International Journal of Applied Biology and Pharmaceutical Technology*.

[68] Nath P., Dhumwad S. D. (2012). Antimicrobial Studies of Co (II), Ni (II), Cu (II) and Zn (II) Complexes Derived from Schiff Bases of 3- Formyl Quinoline and 3-Hydrazinoquinoxalin-2(1H) One. *Rasayan Journal of Chemistry*.

[69] Vivekanand B., Raj M., Mruthyunjayaswamy B. H. M. (2015). Synthesis, Characterization, Antimicrobial, DNA-Cleavage and Antioxidant Activities of 3-((5-Chloro-2-Phenyl-1h-Indol-3-Ylimino)methyl)quinoline-2(1h)-Thione and its Metal Complexes. *Journal of Molecular Structure*.

[70] Numan A. T., Atiyah E. M., Al-Shemary R. K., Abd_Ulrazzaq S. S. (2018). Composition, Characterization and Antibacterial Activity of Mn(II), Co(II),Ni(II), Cu(II) Zn(II) and Cd(II) Mixed Ligand Complexes Schiff Base Derived From Trimethoprim with 8-Hydroxy Quinoline. *Journal of Physics: Conference Series*.

[71] Narayanachar D., Synthesis S. D. (2011). Characterization, Electrochemical, In Vitro Antimicrobial and DNA Cleavage Studies of Co(II), Ni(II), Cu(II) and Zn(II) Complexes of Schiff Bases Derived from 2-Oxo-Quinoline-3-Carbaldehyde. *Main Group Chemistry*.

[72] Meghdadi S., Amirnasr M., Azarkamanzad Z. (2013). Benign Synthesis of the Unsymmetrical Ligand N-(quinolin-8-yl)pyrazine-2-carboxamide. Preparation, Electrochemistry, Antibacterial Activity, and Crystal Structures of Cu(II) and Zn(II) Complexes. *Journal of Coordination Chemistry*.

[73] Al-Hazmi G. A. A., Saad F. A. (2015). A Comparative Antimicrobial Study in Between a Quinoline Drug and its Complexes: Spectral, Kinetic, and Molecular Modeling Investigations. Synthesis and Reactivity in Inorganic, Metal-Organic. *Nano-Metal Chemistry*.

[74] Romanović M. Č., Čobeljić B., Pevec A. (2017). Synthesis, Crystal Structures and Antimicrobial Activity of Azido and Isocyanato Zn(II) Complexes With the Condensation Product of 2-quinolinecarboxaldehyde and Girard’s T Reagent. *Journal of Coordination Chemistry*.

[75] Qin J., Li F. X., Xue L. (2014). Synthesis, Crystal Structures, and Antibacterial Evaluation of Metal Complexes Based on Functionalized 2-phenylquinoline Derivatives. *Acta Chimica Slovenica*.

[76] El-Halim H., Mohamed G. (2017). Synthesis, Spectroscopic, Thermal Analyses, Biological Activity and Molecular Docking Studies on Mixed Ligand Complexes Derived From Schiff Base Ligands and 2,6-pyridine Dicarboxylic Acid: Synthesis, Spectroscopic, Docking Studies on Mixed Ligand Complexes. *Applied Organometallic Chemistry*.

[77] TasminaKhatun M., Alim M., Zahan M. K. E., Haque M., Reza M. (2016). Synthesis and Characterization With Antimicrobial Activity of Ni(II) and Zn(II) Metal Complexes Containing Diphenyl Acetic Acid and Heterocyclic Amine Bases. *Advances in Applied Science Research*.

[78] Patel P. N., Patel K. D., Patel H. S. (2011). Synthesis and Biological Study of Novel 5-{(4-(6,7-Dihydrothieno-[3,2-C]pyridin-5(4h)-Ylsulfonyl)phenylamino)-Methyl}quinolin-8-Ol and its Metal Complexes. *Chinese Chemical Letters*.

[79] Zhang L., Man Z. W., Zhang Y., Hong J., Guo M. R., Qin J. S. (2016). Structure Evaluation, Spectroscopic and Antibacterial Investigation of Metal Complexes With 2-(Pyridin-4-Yl)quinoline-4-Carboxylic Acid. *Acta Chimica Slovenica*.

[80] Jia X., Yang F. F., Li J., Liu J. Y., Xue J. P. (2013). Synthesis and In Vitro Photodynamic Activity of Oligomeric Ethylene Glycol–Quinoline Substituted Zinc(II) Phthalocyanine Derivatives. *Journal of Medicinal Chemistry*.

[81] Mandewale M. C., Thorat B., Nivid Y., Jadhav R., Nagarsekar A., Yamgar R. (2018). Synthesis, Structural Studies and Antituberculosis Evaluation of New Hydrazone Derivatives of Quinoline and Their Zn (II) Complexes. *Journal of Saudi Chemical Society*.

[82] Oliveri V., Grasso G. I., Bellia F., Attanasio F., Viale M., Vecchio G. (2015). Soluble Sugar-Based Quinoline Derivatives as New Antioxidant Modulators of Metal-Induced Amyloid Aggregation. *Inorganic Chemistry*.

[83] Dixit R. B., Patel T. S., Vanparia S. F., Kunjadiya A. P., Keharia H. R., Dixit B. C. (2011). DNA-Binding Interaction Studies of Microwave Assisted Synthesized Sulfonamide Substituted 8-hydroxyquinoline Derivatives. *Scientia Pharmaceutica*.

[84] Huo Y., Wang C., Lu J., Hu S., Li X., Zhang L. (2015). A Novel Trimeric Zn (II) Complex Based on 8-hydroxyquinoline With Trifluoromethylbenzene Group: Synthesis, Crystal Structure, Photophysical Properties and DNA Binding. *Journal of Molecular Structure*.

[85] Wang X. Q., Zhao C. P., Zhong L. C. (2018). Preparation of 4-Flexible Amino-2-Arylethenyl-Quinoline Derivatives as Multi-Target Agents for the Treatment of Alzheimer’s Disease. *Molecules*.

[86] Turnaturi R., Oliveri V., Vecchio G. (2016). Biotin-8-hydroxyquinoline Conjugates and Their Metal Complexes: Exploring the Chemical Properties and the Antioxidant Activity. *Polyhedron*.

[87] Abd El‐Halim H. F., Mohamed G. G. (2018). Synthesis, Spectroscopic, Thermal Analyses, Biological Activity and Molecular Docking Studies on Mixed Ligand Complexes Derived from Schiff Base Ligands and 2, 6‐pyridine Dicarboxylic Acid. *Applied Organometallic Chemistry*.

[88] El-Remaily M. A. E. A. A. A., Abu-Dief A. M., Elhady O. (2019). Green Synthesis of TiO2 Nanoparticles as an Efficient Heterogeneous Catalyst with High Reusability for Synthesis of 1,2-dihydroquinoline Derivatives. *Applied Organometallic Chemistry*.

[89] Numan A. T., Al-Taweel H. H., Ganawi A. M. (2015). Synthesis and Characterization of Some Biological Active Transition Metal Complexes of Schiff Base Derived from Cefixime With Mixed Ligand 8-hydroxy Quinoline. *Baghdad Science Journal*.

[90] Pavlidis N., Kofinas A., Papanikolaou M. G. (2021). Synthesis, Characterization and Pharmacological Evaluation of Quinoline Derivatives and Their Complexes With Copper(ΙΙ) in In Vitro Cell Models of Alzheimer’s Disease. *Journal of Inorganic Biochemistry*.

[91] Mandewale M. C., Thorat B. R., Yamgar R. S. (2015). Synthesis and Anti-Mycobacterium Study of Some Fluorine Containing Schiff Bases of Quinoline and Their Metal Complexes. *Der Pharma Chemica*.

[92] Bacchi A., Carcelli M., Compari C. (2011). Investigating the Role of Metal Chelation in HIV-1 Integrase Strand Transfer Inhibitors. *Journal of Medicinal Chemistry*.

[93] Ahmed H. M. A. E. L., Ali M. M. K., El-Dabea T., Alzahrani S., Abu-Dief A. M. (2024). Nano-sized Mixed Ligand [4-Bromo-2-(quinolin-2-Yliminomethyl)-Phenol Imine-Phenanthroline] Ru (III) Complex for Medicinal Applications.

